# Spatial and single-cell profiling of the metabolome, transcriptome and epigenome of the aging mouse liver

**DOI:** 10.1038/s43587-023-00513-y

**Published:** 2023-11-09

**Authors:** Chrysa Nikopoulou, Niklas Kleinenkuhnen, Swati Parekh, Tonantzi Sandoval, Christoph Ziegenhain, Farina Schneider, Patrick Giavalisco, Kat-Folz Donahue, Anna Juliane Vesting, Marcel Kirchner, Mihaela Bozukova, Christian Vossen, Janine Altmüller, Thomas Wunderlich, Rickard Sandberg, Vangelis Kondylis, Achim Tresch, Peter Tessarz

**Affiliations:** 1 Max Planck Research Group ‘Chromatin and Ageing’, Max Planck Institute for Biology of Ageing, Cologne, Germany; 2 Cologne Excellence Cluster on Cellular Stress Responses in Aging-associated Diseases (CECAD), Cologne, Germany; 3 Institute of Medical Statistics and Computational Biology, Faculty of Medicine, University of Cologne, Cologne, Germany; 4 Department of Cell and Molecular Biology, Karolinska Institutet, Stockholm, Sweden; 5 Institute for Pathology, University Hospital Cologne, Cologne, Germany; 6 Metabolic Core Facility, Max Planck Institute for Biology of Ageing, Cologne, Germany; 7 FACS and Imaging Core Facility, Max Planck Institute for Biology of Ageing, Cologne, Germany; 8 Max Planck Institute for Metabolism Research, Cologne, Germany; 9 Cologne Center for Genomics, University of Cologne, Cologne, Germany; Berlin Institute of Health at Charité, Core Facility Genomics, Berlin, Germany; Max Delbrück Center for Molecular Medicine in the Helmholtz Association, Berlin, Germany; 10 Department of Gastroenterology, Hepatology and Infectious Diseases, University Hospital Düsseldorf, Medical Faculty at Heinrich-Heine-University, Duesseldorf, Germany; 11 Present Address: Global Computational Biology and Digital Sciences, Boehringer Ingelheim Pharma, Biberach, Germany; 12 Present Address: Department of Human Biology, Faculty of Science, Radboud Institute for Molecular Life Sciences, Radboud University, Nijmegen, The Netherlands

**Keywords:** Chromatin structure, Transcription, Lipidomics, Data processing, Ageing

## Abstract

Tissues within an organism and even cell types within a tissue can age with different velocities. However, it is unclear whether cells of one type experience different aging trajectories within a tissue depending on their spatial location. Here, we used spatial transcriptomics in combination with single-cell ATAC-seq and RNA-seq, lipidomics and functional assays to address how cells in the male murine liver are affected by age-related changes in the microenvironment. Integration of the datasets revealed zonation-specific and age-related changes in metabolic states, the epigenome and transcriptome. The epigenome changed in a zonation-dependent manner and functionally, periportal hepatocytes were characterized by decreased mitochondrial fitness, whereas pericentral hepatocytes accumulated large lipid droplets. Together, we provide evidence that changing microenvironments within a tissue exert strong influences on their resident cells that can shape epigenetic, metabolic and phenotypic outputs.

## Abstract (web summary)

Using spatial and single-cell multiomics, Nikopoulou et al analyze how different cells within the mouse liver age, revealing zonation-specific aging trajectories and highlighting the importance of the local tissue microenvironment.

## Main

Aging is characterized by a general physiological decline that is accompanied by metabolic, epigenetic and transcriptional changes^1^. A common attribute for these alterations is an increased inter-individual heterogeneity as observed in large cohorts. Even on an organismal level within populations of genetically identical individuals, variability seems intrinsically interconnected with aging. For example, in cohorts of *Caenorhabditis elegans* or mice, some individuals die much earlier than others^2^.

It is largely appreciated that transcriptional variability increases with age^3–5^. Although whole-tissue omics approaches have been important to get an insight into the uniform changes that occur on the organ level during aging, such methods cannot investigate heterogeneity on a cellular level. It is therefore unresolved whether all cells of the same cell type in a tissue age in the same way or whether the location of the cells within a tissue matters in this context. The development of single-cell and spatial omics methods renders it now possible to obtain (spatially resolved) molecular profiles at close to single-cell resolution, thus providing promising tools for deciphering the multifaceted process of aging^6^.

The liver is a heterogeneous tissue that consists of hepatocytes arranged in repeating units of hexagonally shaped lobules. Blood flows into the lobule from portal veins and hepatic arteries at the corners of the lobules to the central veins. This architecture creates gradients of oxygen, nutrients and hormones^7^. This gradual change in the lobule’s microenvironment is also referred to as liver zonation^8^, and the resulting spatial division of labor is essential for the optimal function of the liver. For example, the outer highly oxygenated periportal lobule layers perform mitochondrial-dependent metabolic tasks such as *β*-oxidation whereas the low oxygen concentrations at the pericentral areas will drive glycolysis^7^. As hepatocytes are the primary cells that perform these metabolic processes and their metabolic characteristics depend on location, the liver is an attractive tissue to address the impact of location and metabolic state on the aging trajectory within a dedicated cell type.

Here, we used spatial transcriptomics, single-cell assay for transposase-accessible chromatin with sequencing (scATAC-seq) and single-cell RNA sequencing (scRNA-seq) in combination with lipidomics and functional assays for mitochondrial activity to reveal zonation-specific patterns of hepatocyte aging. An obvious phenotypic difference between the young and aging liver is the deposition of fat, which is concentrated mainly around the central vein. Using spatial transcriptomics, we gain insight into this phenotype’s molecular underpinnings by identifying genes involved in lipid biosynthetic pathways. On the other hand, the most substantial age-related changes in the periportal region are associated with mitochondrial dysfunction. Zonation and age are important axes of separation in single-cell ATAC data, indicating that location and organismal aging are major drivers of epigenomic changes in the liver. Using scRNA-seq on sorted hepatocytes, we show that transcriptional noise is buffered by an increase in ploidy. The data presented here shed light on age-related changes in liver tissue microenvironments and will serve as a resource for the hepatic and aging community.

## Results

### Spatial transcriptomics gives insights into zonation-specific and age-related metabolic rearrangements

**Fig. 1:**
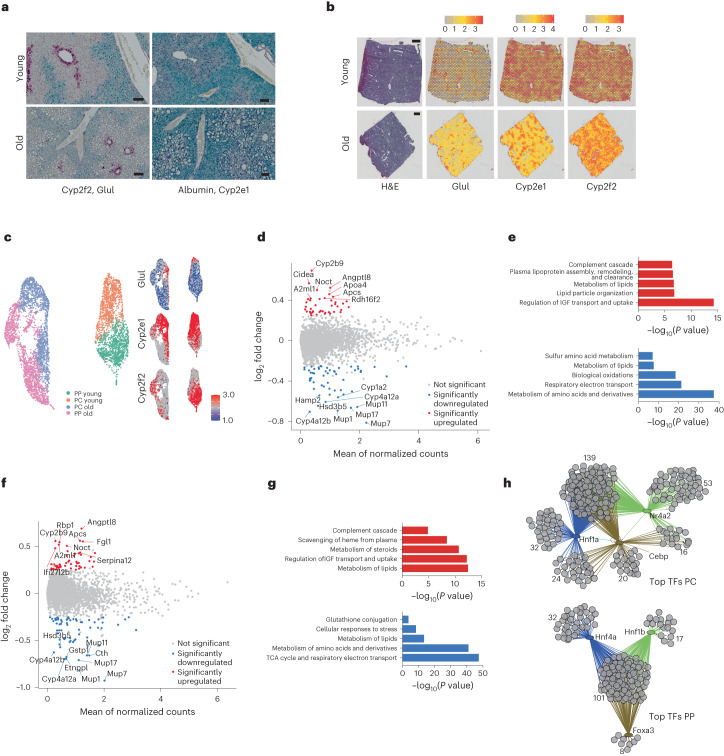
Age-related and zonation-specific transcriptional alterations. **a**, RNAscope of zone-specific marker genes Glul (magenta, upper panel), Cyp2f2 (cyan, upper panel), Cyp2e1 (magenta, lower panel) and albumin (cyan, lower panel) in paraffin-embedded liver sections from young (3-month-old) and old (18-month-old) mice. Scale bars, 100 µm. **b**, H&E staining of one young (upper panel) and one old (lower panel) liver specimen used for spatial transcriptomics (scale bar, 500 µm) and plots showing the expression levels of Glul, Cyp2f2 and Cyp2e1 indicated by color. The color gradient represents normalized gene expression. **c**, UMAP projection of the spatial data; color-coded are the different zones and ages (left panel) and the expression of Glul, Cyp2e1 and Cyp2f2 (right panel). PP: periportal, PC: pericentral. **d**, MA ((M (log2 ratio) and A (mean average)) plots of gene expression changes upon aging in the pericentral zone. Significantly changed genes are colored in red and blue (based on MAST^18^). Bonferroni correction was applied for multiple testing adjustments of *P* values (threshold of 0.05). **e**, Top five Reactome pathways for up- and downregulated genes in the pericentral region analyzed using Metascape. **f**, MA plots of gene expression changes upon aging in the periportal zone. Significantly changed genes were colored in and blue (based on MAST^18^). Bonferroni correction was applied for multiple testing adjustments of *P* values (threshold of 0.05). **g**, Top five Reactome pathways for up- and downregulated genes in the periportal region analyzed using Metascape. **h**, Transcription factor (TF) activity prediction from the age-dependent differentially expressed genes by the iRegulon app in Cytoscape (based on Supplementary Table 3; see Methods for details). For each zone, the top predicted transcription factors are shown as well as their interaction to regulate transcripts. Numbers indicate the genes in every cluster.

**Extended Data Fig. 1:**
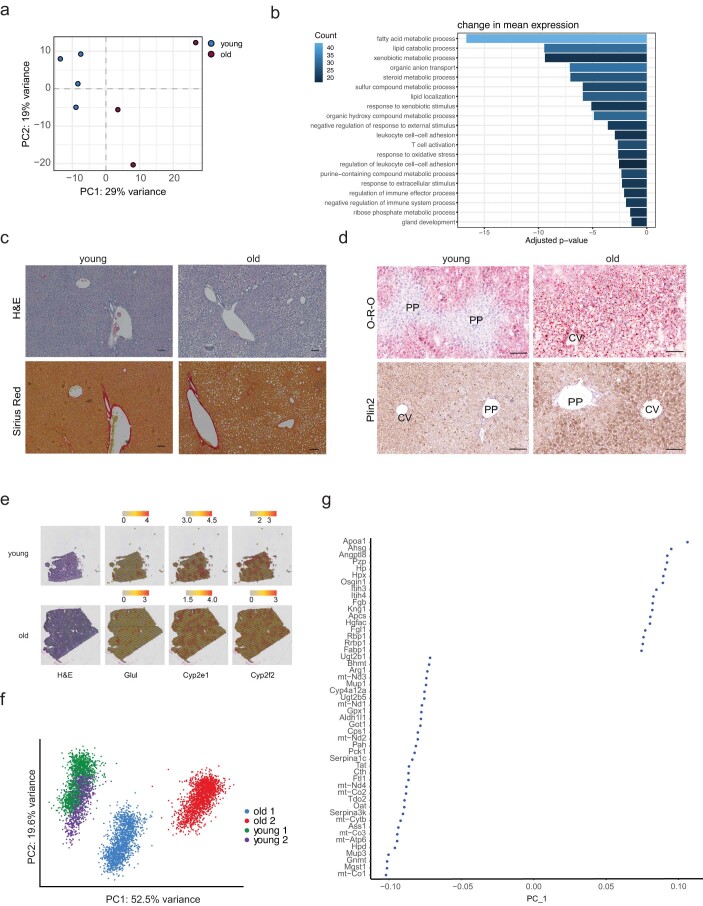
Lipid Remodeling in the ageing mouse liver. **a**) PCA projection of bulk RNA-seq data^11^ derived from young and old mouse livers. **b**) Differentially enriched Gene Ontology Biological Processes in the aged liver tissue derived from A (Supplementary Table 1). The colour scale represents the number of genes in each term and Adjusted p-value (Benjamini Hochberg) on x-axis is derived with over representation test using enrichGO function of clusterProfiler R package. **c**) Representative images from H&E (upper panel) and Sirius Red (lower panel) stainings on liver sections from a young and an old mouse. Scale bar = 100μm. **d**) Representative images of PP (periportal) and CV (central vein areas) of Oil-red-O (O-R-O, upper panel) and Plin2 immunostainings (lower panel) on liver sections from young and old mice. Scale bar = 100 µm. **e**) Spatial transcriptomics slides of the second biological replicate. **f**) PCA plot of the spatial data after integration of the four datasets using canonical correlation analysis. Different colours represent the different samples. **g**) PC plot showing the top 50 genes that separate the ageing groups in Supplementary Fig. 1e.

Transcriptional profiling of the mouse liver has revealed alterations in metabolic pathways^9–11^, with a major fraction of genes contributing to alterations in lipid metabolism (Extended Data Fig. 1a,b and Supplementary Table 1). Changes in lipid metabolism have been described to occur during aging and recently lipidomics started to identify corresponding changes in lipid profiles^12^. Liver pathologies that involve fat deposition, such as nonalcoholic fatty liver disease (NAFLD) show a tendency towards zonated lipid deposition around the central area^13^, but we were not aware of any dataset investigating lipid deposition in the aging liver with respect to the specific zones. To assess the lipid deposition around the main zones, we performed RNAScope for pericentral (Cyp2e1, Glul) and periportal markers (Albumin, Cyp2f2) (ref. ^14^) combined with hematoxylin and eosin (H&E) staining in liver isolated from young (3–4 months) and old (18–22 months) mice (Fig. 1a and Extended Data Fig. 1c). Importantly, Sirius red staining showed no profound increase in liver fibrosis in old livers (Extended Data Fig. 1c). On the contrary, oil red O (O-R-O) staining (Extended Data Fig. 1d, upper panel) and immunohistochemical (IHC) staining for PLIN2 (Extended Data Fig. 1d, lower panel), a protein known to be enriched at the outer membrane of lipid droplets (LDs)^15^, showed that large LDs accumulate around the central vein in aged livers.

**Extended Data Fig. 2:**
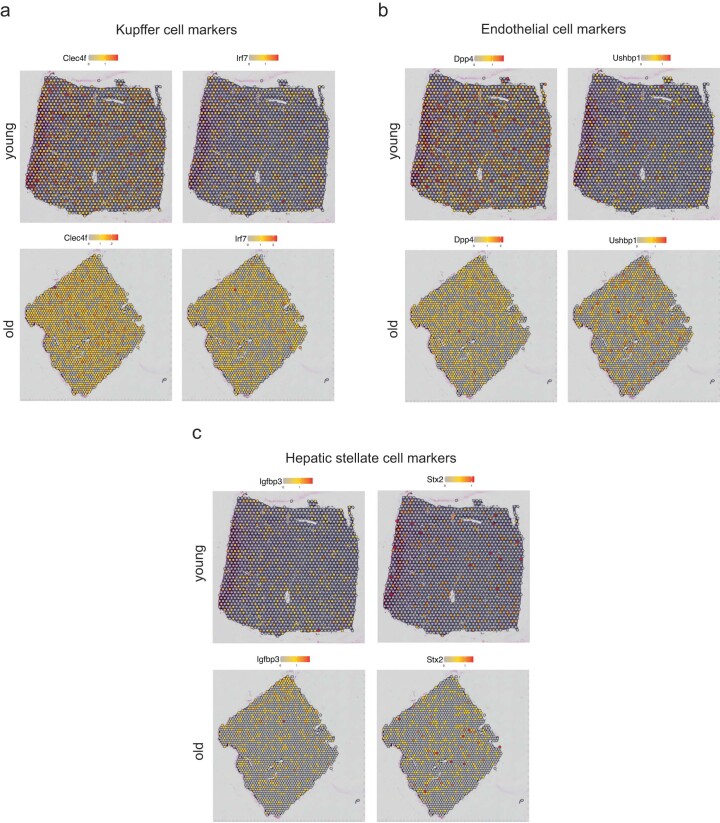
Non-parenchymal cells in the spatial transcriptomics data. Representative plots showing expression levels of Kupffer cell (**a**), endothelial cell (**b**) and hepatic stellate cell (**c**) markers as indicated in young and old livers as determined by spatial transcriptomics. The colour gradient represents normalised gene expression.

The apparent zone-dependent deposition of lipids in the aging liver prompted us to investigate the underlying transcriptional events. We used the 10X Genomics Visium Platform and ran 10 µm tissue cryosections from livers of two young and two old mice (see Supplementary Table 2 for sequencing metrics). Initially, we visualized the normalized spatial gene expression of the zonation markers *Cyp2f2, Cyp2e1* and *Glul* in young and old liver (Fig. 1b and Extended Data Fig. 1e). Based on the spatial expression patterns of these marker genes, we were able to resolve pericentral and -portal areas. Principal Components analysis showed that spots from the two young liver slides cluster together, while spots from the two old slides separate (Extended Data Fig. 1f). We merged young and old samples individually using canonical correlation analysis^16^ to remove batch effects. Subsequently, we analyzed zonal expression effects. Then, we merged all datasets using the same strategy. To assess whether the sample separation reflected gene expression differences based on age or were mostly due to a potential batch effect, we used the loadings calculated in the principal-component analysis (PCA) and intersected those with a recently published resource, in which global aging genes were defined organismal and tissue-wide^17^. Of the top 50 genes that contributed to the first principal component, the majority (35/50) were part of the liver-specific global aging genes (Extended Data Fig. 1g), indicating that our analysis preserved age-related expression differences. Next, we assigned spots based on *Cyp2e1* and *Cyp2f2* expression levels to mark pericentral and periportal region, respectively (Fig. 1c and Methods). Finally, we performed differential expression analysis based on a two-part, generalized linear hurdle model^18^ (Fig. 1d,f). Overall, 429 genes were differentially expressed in the aged pericentral zone and 544 in the periportal zone. 375 genes were commonly deregulated in both zones (Supplementary Table 3). To understand which biological processes were affected most by age and zonation, we performed pathway enrichment using Metascape^19^ (Supplementary Table 3). Reactome pathways indicated that amino acid metabolism, lipid metabolic processes and mitochondrial energy production were downregulated, while complement cascade and IGF transport and uptake was upregulated in both zones (Fig. 1e,g). Specific to the pericentral zones was an upregulation of plasma lipoprotein assembly, remodeling and clearance as well as lipid particle organization (Fig. 1e). The periportal zone was characterized by an upregulation of heme scavenging and a down-regulation of stress signaling pathways (Fig. 1g). Important to note is the observation that while hepatocyte are dominating the transcriptional profiles in the spatial dataset, underlying marker gene expression for other cell types suggest that this dataset might be used to interrogate non-parenchymal cells, particularly Kupffer, endothelial and stellate cells (Extended Data Fig. 2a–c). Finally, we wanted to understand whether the transcriptional changes were driven by a dedicated set of transcription factors. We used the iRegulon app within Cytoscape^20,21^ and visualized the top three most significant TFs (normalized enrichment score > 4) based on age-dependent differential expression within the two zones. Shared between the zones is Hnf1, which has been shown to regulate many hepatic genes^22^. Genes in the periportal area were predicted to be regulated by Hnf4a and Foxa3 (Fig. 1h). Hnf4a is a master regulator during hepatic differentiation and plays an important role during liver regeneration^23^, similarly to Foxa3^24^. In addition, Hnf4a has recently been shown to possess anti-proliferative capacity and thus protects against hepatocellular carcinoma^23^. On the other hand, genes in the pericentral zone were predicted to be regulated by Cebp and Nr4a2 (Fig. 1h), two TFs that regulate glucose and lipid metabolism^25,26^. Taken together, spatial transcriptomics revealed that aging is accompanied by zonation-specific metabolic rewiring, which is driven by a network of dedicated transcription factors.

### The aging liver is characterized by lipid remodeling and loss of SRC in periportal mitochondria

**Fig. 2:**
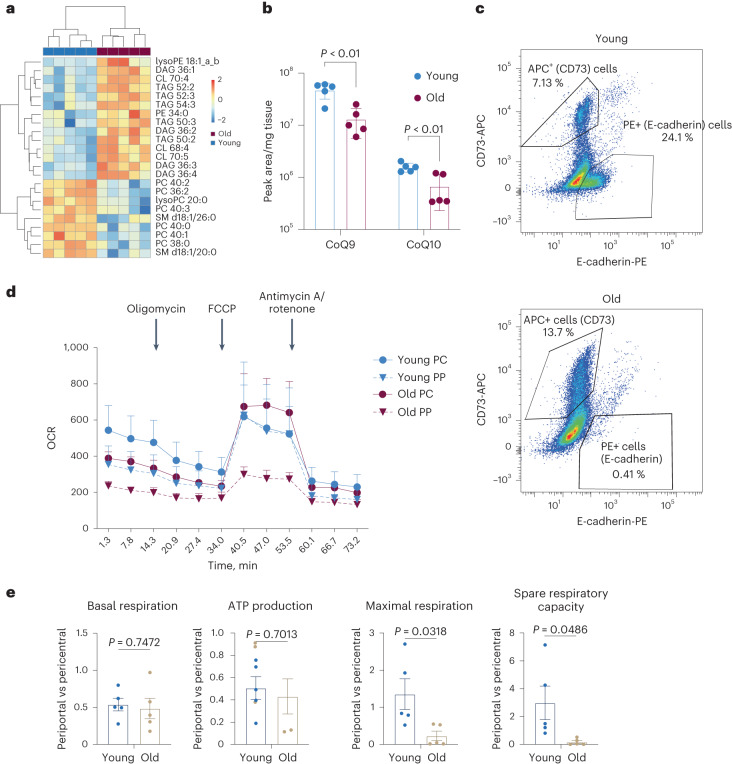
Lipid remodeling and alterations in mitochondrial metabolism in the aging liver. **a**, Heatmap with hierarchical clustering of lipid datasets derived from five old and five young mouse livers, showing the differentially regulated classes of lipids. Hierarchical clustering was performed using LipidSig based on data available in Supplementary Table 4. **b**, Bar plot showing the expression of Ubiquinones CoQ9 and CoQ10 in young (*n* = 5) and old (*n* = 5) liver. Data are presented as mean values ± standard error of the mean (s.e.m.). Statistical significance was determined using an unpaired two-tailed *t*-test. **c**, Exemplary FACS profiles of sorted hepatocytes based on CD73 (pericentral) and E-cadherin (periportal). **d**, Seahorse profile of hepatocytes purified from indicated sources. Error bars represent s.e.m. from *n* = 5 (*n* represents data derived from individual animals). **e**, Mitochondrial function as measured by Seahorse Mitochondrial Stress kit (parameter on top of graph) expressed as periportal versus pericentral and young-old *n* = 5 (*n* represents data derived from individual animals). Error bars represent s.e.m. Statistical significance was determined using a two-tailed unpaired *t*-test. Source data

**Extended Data Fig. 3:**
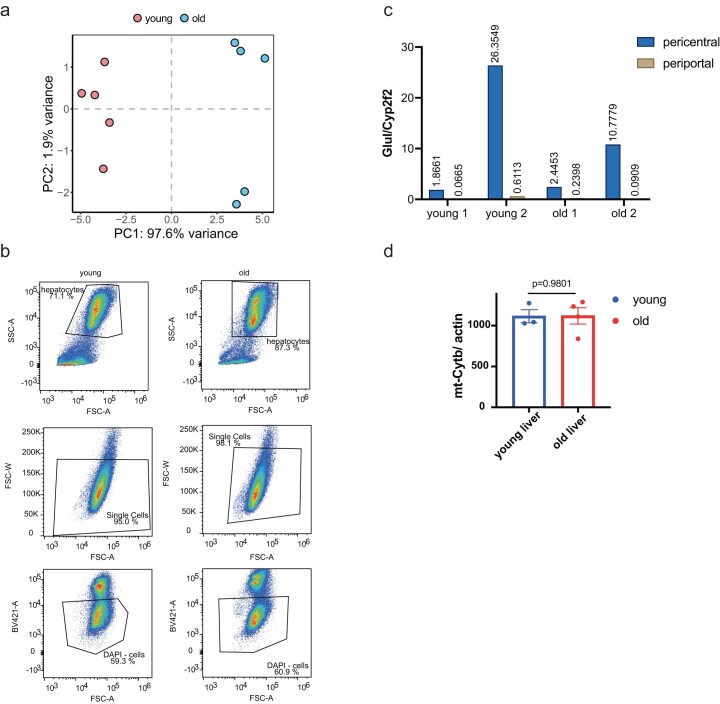
Lipids and sorting of hepatocytes based on zones. **a**) PCA of lipidomic data coloured by age. **b**) Gating strategy for isolation of pericentral and periportal hepatocytes. **c**) qRT-PCR to validate the enrichment for pericentral and periportal hepatocytes based on expression ratios of Glul and Cyp2f2 levels. Shown are individual replicates for young and old mice (as indicated). **d**) Mitochondrial content of livers was measured using primers against genomic copies of mt-Cytb and b-actin. Individual values are given as dots. Error bars represent SEM. Statistical significance was determined using a two-tailed unpaired *t*-test.

The spatial transcriptomic data suggested age-related metabolic alterations that depend on the location of cells with respect to central or portal regions. To gain more insight into the metabolic alterations, we first performed lipidomics to characterize the changes in lipid metabolism within the aging liver (Supplementary Table 4). This approach allowed us to not only address storage and membrane lipids but also analyze levels of cardiolipins (CLs) and ubiquinones to further investigate the observed alterations in mitochondrial metabolism. We extracted lipids from the livers of young and old mice. PCA (Extended Data Fig. 3a) suggested a strong lipid remodeling for most of the major lipid classes. Hierarchical clustering using LipidSig^27^ showed that long-chained polyunsaturated fatty acids in the form of triacylglyerides were increased in the aging liver as were several diacylglyerides (DAGs), in line with the increase in LDs. Conversely, levels of phosphatidylcholine (PC) and sphingomylein (SM) were decreased (Fig. 2a). Interestingly, an increase in DAGs as well as a decrease in SMs has been linked to reduced insulin insensitivity^28^, a well-known hallmark of aging and a pathway that was also evident in the spatial transcriptomics data (Fig. 1d,e). Another class of lipids that showed increased levels upon aging were CLs (Fig. 2a), which indicated changes in the composition of mitochondrial membranes and hence the function of mitochondrial inner membrane proteins, including the electron transport chain^29^. This hypothesis was also supported by the observation that ubiquinones, lipids that transfer the electron between the different complexes of the electron transport chain, were strongly downregulated with age (Fig. 2b). These findings in combination with the spatial transcriptomics data supported the hypothesis of age-dependent mitochondrial changes. As the spatial transcriptomic data and the lipidome analysis pointed towards a strong impact on mitochondrial metabolism, we wanted to investigate this phenotype in more detail, particularly in a zone-specific manner. In order to do this, we used a previously published protocol^30^ to sort hepatocytes into pericentral and periportal upon perfusion of the liver (Fig. 2c and Extended Data Fig. 3b). This approach depends on the zonation-dependent expression of E-cadherin (periportal) and CD73 (Nt5e, pericentral)^30^ and was able to separate pericentral and periportal hepatocytes as judged by expression of Glul and Cyp2f2 (Extended Data Fig. 3c). Intriguingly, aging was accompanied by a strong decrease in E-Cadherin (Fig. 2c), which might indicate changes in cell-to-cell adhesion and communication. To address mitochondrial function, we first measured mitochondrial content which was variable across different animals, but largely unaltered with age (Extended Data Fig. 3d). Finally, we performed Seahorse analysis using the mitochondrial stress kit to assess mitochondrial function. While basal respiration and ATP production changed only mildly with age (Fig. 2d,e), we observed a striking reduction in the maximal and thus, spare respiratory capacity (SRC) in periportal hepatocytes (Fig. 2d,e). On the other hand, pericentral hepatocytes showed no difference in maximal respiration upon aging. Loss of SRC sensitizes the cells to surges in ATP demand^31^, and it has been proposed that SRC can be used as a measure of mitochondrial health^32^. Taken together, spatial data, lipidomics and bioenergetics measurements point toward an age-dependent decrease in hepatic mitochondrial fitness and function, specifically in the periportal zone of the liver.

### Chromatin accessibility in mouse liver carries a hepatocyte aging signature

**Fig. 3:**
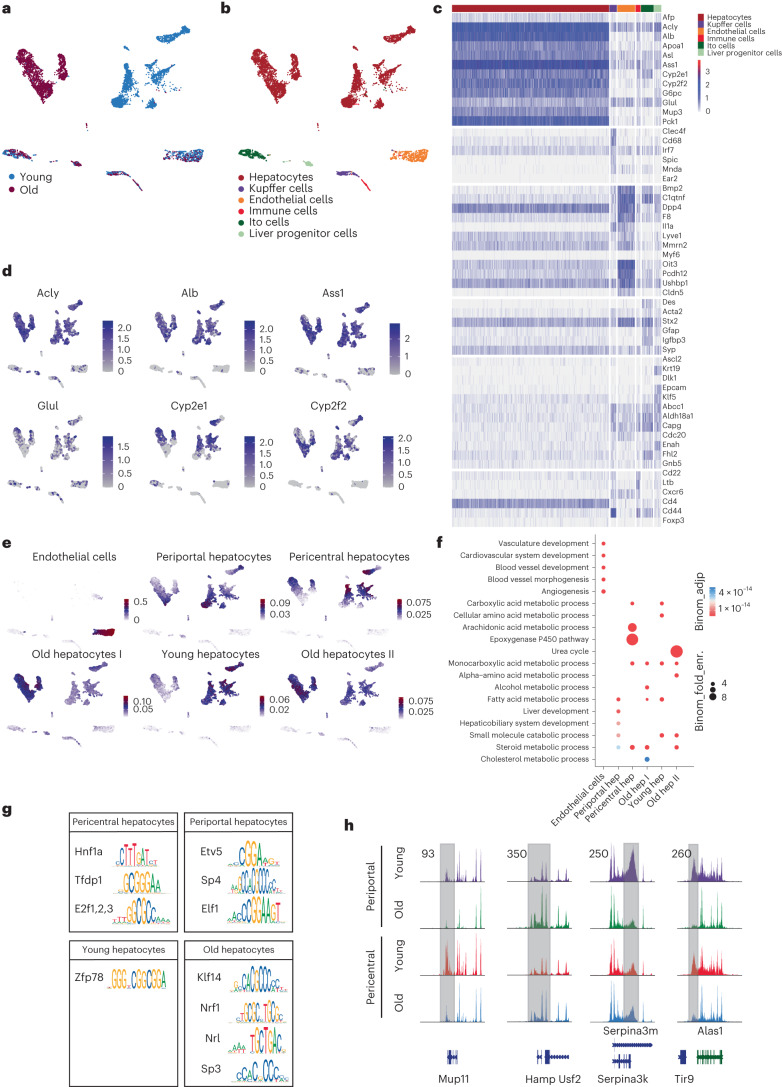
Differential chromatin accessibility in aged liver hepatocytes. **a,b**, UMAP projection of scATAC-seq data of mouse liver nuclei. **a**, Different colors represent liver cells from young and old age groups identified using cisTopic. **b**, Different colors represent different cell types based on imputed marker gene activity. **c**, Heatmap showing the accessibility of indicated marker gene promoters used to call cell types. **d**, Examples of hepatic marker genes and the respective accessibility at their promoters. **e**, Examples of topics as identified by CisTopic (for details, see text). Color code of the UMAPs is according to the normalized topic score for each cell. **f**, GO term analysis of the highlighted topics as shown in **e**. hep: hepatocyte; Binom_fold_enr.: binomial fold enrichment; Binom_adjp: adjusted p-value. Significance threshold was set at 0.05. **g**, Uniquely enriched transcription factors and their corresponding motifs for the highlighted zones/age groups. **h**, Exemplary tracks of differentially accessible sites between pericentral and periportal hepatocytes upon aging. The gray bar indicates altered regions.

**Extended Data Fig. 4:**
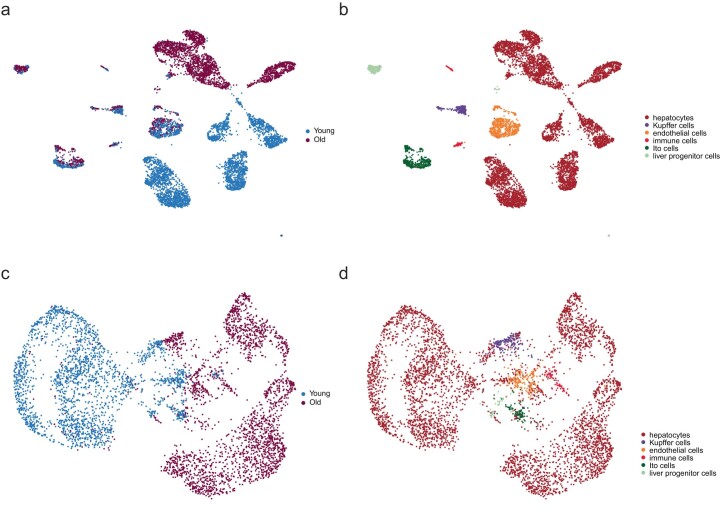
Initial analysis of the individual scATAC-seq samples using Signac. **a**) UMAP projection of scATAC-seq nuclei from young and old livers of biological replicate 1 generated with 10X scATAC v2 chemistry. Colour-coded are the different age groups identified using Signac. **b**) Same as in **a**). Colour coded are the different cell types, assigned by using marker genes from CellMarker. **c**) UMAP projection of scATAC-seq nuclei from young and old livers of biological replicate 2 generated with 10X scATAC v3 chemistry. Colour-coded are the different age groups identified using Signac. **d**) Same as in **b**). Colour coded are the different cell types, assigned by using marker genes from CellMarker.

**Extended Data Fig. 5:**
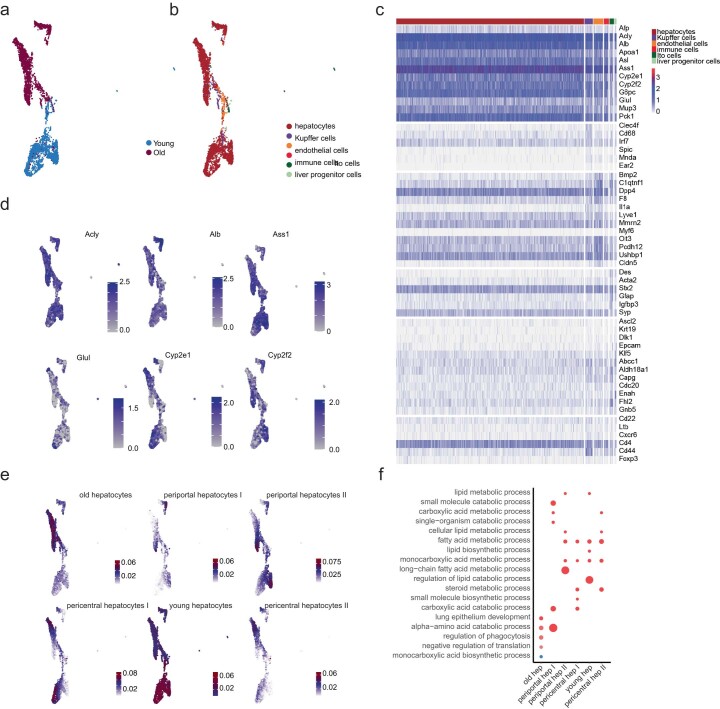
Second scATAC replicate. **a**–**b**) UMAP projection of scATAC-seq data of mouse liver nuclei (2nd biological replicate) **a**) Different colours represent liver cells from young and old age groups identified using cisTopic. **b**) Different colours represent different cell types based on imputed marker gene activity (see also Supplementary Fig. 3c). **c**) Heatmap showing the accessibility of indicated marker gene promoters used to call cell types. **d**) Examples of hepatic marker genes and the respective accessibility at their promoters. **e**) Examples of topics as identified by CisTopic - for details see text. Colour code of the UMAPs is according to the normalised topic score for each cell. **f**) GO term analysis of the highlighted topics.

**Extended Data Fig. 6:**
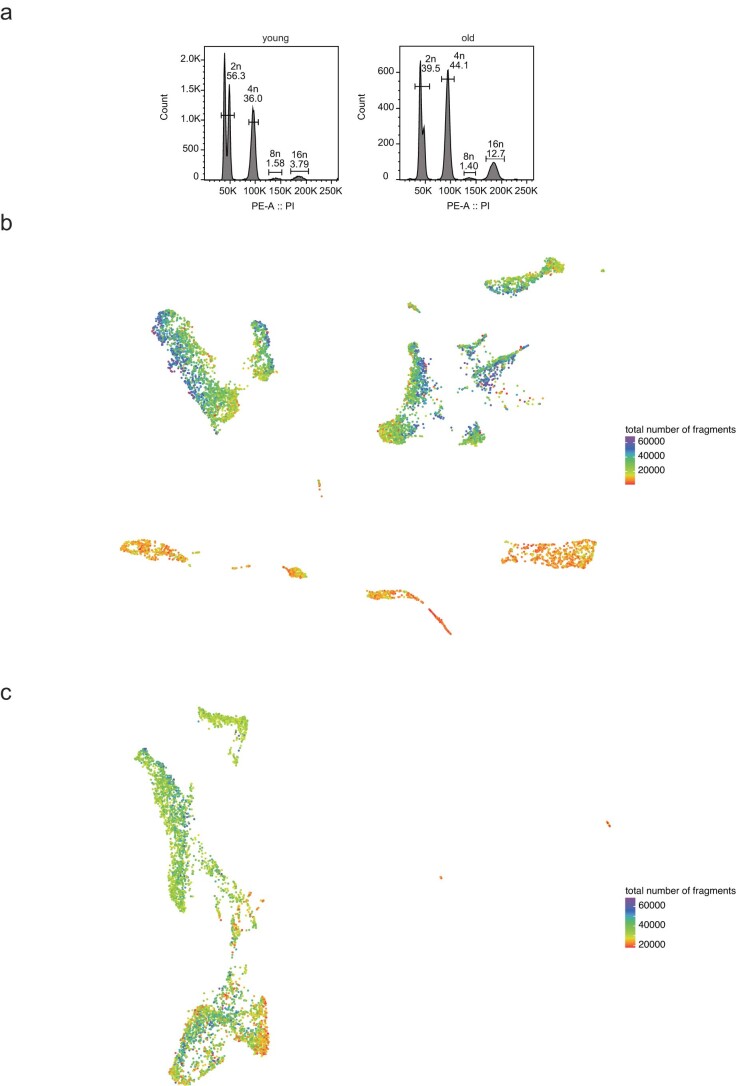
scATAC and ploidy levels. **a**) DNA content of nuclei purified from liver tissue following the same protocol as for scATAC-seq. **b**) **c**) Sequence coverage of individual cells.

Having defined the transcriptional, lipid and functional alterations that occur within the periportal and pericentral zones of the aging liver, we next wanted to investigate if epigenetic changes underlie these differences. Therefore, we performed scATAC-seq using the 10x Chromium platform. In two independent experiments, we profiled a total of 6,579 nuclei (4,320/2,259) prepared from young liver tissue and 5750 nuclei (3,260/2,490) from old liver tissue. For each experiment, the purified liver nuclei of four mice were mixed 1:1:1:1 prior tagmentation. Sequencing metrics can be found in Supplementary Table 2, and the initial analysis and comparison of the two individual biological replicates using Signac^33^ can be found in Extended Data Fig. 4. In both independent datasets, we identified several clusters indicating that we profiled different liver-resident cells. Interestingly, all smaller clusters showed integration of young and old cells, suggesting that their epigenome did not change dramatically with age. On the other hand, the larger clusters clearly separated young and old tissue, indicating that their epigenome changed significantly with age. For further analysis, we turned to CisTopic^34^ to identify cluster-specific regions in the genome that might represent aging signatures. Projecting cells in a UMAP based on CisTopic-mediated clustering confirmed the separation of the large cluster depending on age (Fig. 3a and Extended Data Fig. 5a). To identify cell types, we inferred transcriptional activity from the respective promoter accessibility, as described previously^35^. We used known marker genes^14,36^ and CellMarker (http://bio-bigdata.hrbmu.edu.cn/CellMarker/) to infer the cellular identity of each cluster, which enabled us to resolve all expected cell types of the liver, except for cholangiocytes (Fig. 3b,c and Extended Data Fig. 5b,c). We were not able to distinguish different immune cell types, as their marker genes’ imputed activity was ambiguous (Fig. 3b,c and Extended Data Fig. 5b,c). In line with the observation that the livers were not fibrotic, we did not observe a notable increase in immune or hepatic stellate cells. Notably, young and old cells of one type mapped well into the same cluster for all but the hepatocytes. We conclude that epigenomic changes in hepatocytes are more pronounced than in the other detected cell types (Fig. 3a,b and Extended Data Fig. 5a,b). It is well established that the aging liver accumulates polyploid hepatocytes^37^. Fluorescence-activated cell sorting (FACS) analysis of nuclei obtained by our scATAC nuclear isolation protocol found nuclei of 2n up to 16n ploidy (Extended Data Fig. 6a). Consistent with previous findings^37^, the rate of polyploidy was higher in old cells (60.5%) than in young cells (43.7%). Higher ploidy levels might increase coverage in the scATAC-seq profiles and confound age or cell type-related differences. Because, to the best of our knowledge, it is not possible to infer ploidy levels from scATAC data, we took coverage as a proxy. We observed a substantially higher coverage in hepatocytes than in other cell types (Extended Data Fig. 6b,c). Yet, coverage levels in the old and the young hepatocytes clusters were comparable and most likely not a cause for the age separation of hepatocytes.

To check whether liver zonation is reflected in scATAC profiles as well, we plotted the imputed gene activity of the known zonation markers *Glul, Cyp2e1* and *Cyp2f2* (Fig. 3d and Extended Data Fig. 5d; compare to general hepatocyte markers *Acly, Ass1* and *Alb*). Their activity is highest in the hepatocyte clusters, and the activity patterns of the pericentral markers *Glul* and *Cyp2e1* are complementary to that of *Cyp2f2*. As we were specifically interested in understanding whether there were any dedicated profiles in hepatocytes with respect to aging and/or zonation, we made use of the inferred *cis*-regulatory topics that underlie the latent Dirichlet allocation (LDA) used by cisTopic^34^ and transferred the topic assignments to the individual clusters. These topics are defined by specific accessible regions in the genome, thus factoring in open promoters, enhancers and transcription factor motifs. Thus, topics can be for instance used to predict cell types^34^. Indeed, using endothelial cells as an example, the topic probability highlights this cluster (Fig. 3e). The corresponding gene signature and its corresponding pathways can also be visualized using Gene Ontology (GO) terms, which highlights the role of endothelial cells in the vascular system (Fig. 3f). Using the identical approach and rationale on hepatocytes, it became apparent that age and zonation were predicted to belong to different topics (Fig. 3e and Extended Data Fig. 5e). This finding supported our hypothesis that the different zones in the liver had different impacts on the age-related epigenetic changes. On a pathway level, fatty and amino acid metabolic pathways were differentially regulated between the different topics within the hepatocytes (Fig. 3f and Extended Data Fig. 5f). Finally, we wanted to investigate whether there were specific transcription factors underlying the differences in topic distribution using Rcistarget^38^. The difference in metabolic tasks is also evident in the transcription factors predicted based on topic-defining regions. Hnf1a has been reported to affect cell lineage differentiation, lipid metabolism, glucose metabolism^39^ and E2f is involved in cell cycle progression, but have also been linked to glucose metabolism^40^. On the other hand, Etv5 is involved in fatty acid metabolism^41^. Comparing young to aged hepatocytes, Nrf1 was predicted to be enriched in old animals. Interestingly, Nrf1 has been shown to be important for protection against oxidative stress^42^, a pathway strongly linked with the aging process (Fig. 3g). Together, the data indicated that also on an epigenetic level, hepatocytes are clearly separated into pericentral and periportal, which can also be visualized on a pseudo-bulk level (Fig. 3h).

### Specific Cidea expression in the pericentral zone is driven by chromatin architectural changes

**Fig. 4:**
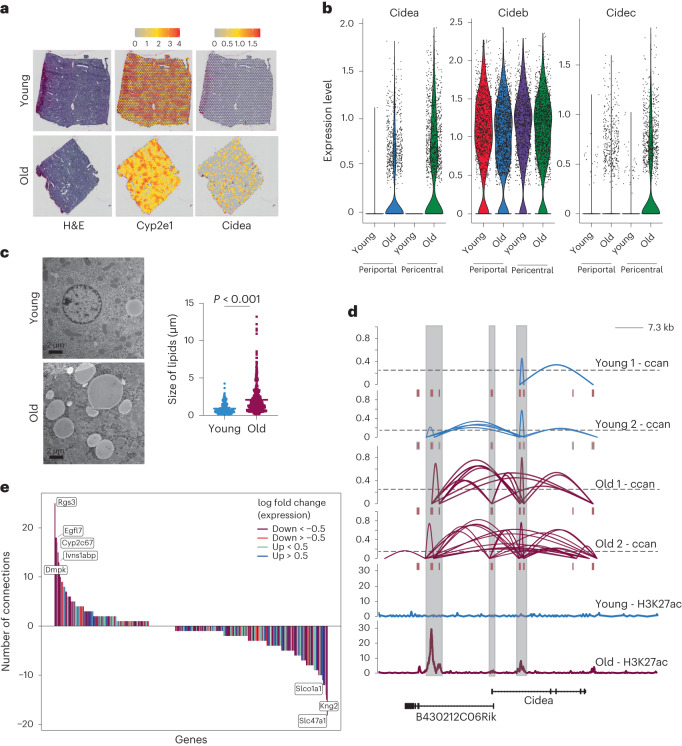
Connection between chromatin and transcriptional alterations in the aging liver. **a**, H&E staining of one young (upper panel) and one old (lower panel) liver specimen used for spatial transcriptomics and a plot showing the expression level of Cidea. Please note that H&E stain and Cyp2e1 plots are identical to Fig. 1b and used here for reference only. The color gradient represents normalized gene expression. **b,** Violin plots indicating the expression levels of Cidea, Cideb and Cidec across pericentral and periportal regions in young and old liver. **c**, Transmission electron micrograph of LDs of young and old liver tissue. Representative images at 3,000×; scale bars, 2 µm. ImageJ quantification of the mean LD diameter size (μm) from ten randomly selected photos from 5 young (LD *n* = 327, mean = 0.9053) and 4 old (LD *n* = 407, mean = 2.084) mouse specimens. Statistical significance was determined using an unpaired two-tailed *t*-test. **d,** Ccan values based on Cicero^47^ prediction of co-accessibility (upper panel) and the enhancer mark H3K27ac (lower panel) at the Cidea locus in young and old mouse liver. Highlighted in gray are potential enhancer and promoter regions from Cidea and its associated antisense long non-coding RNA, respectively. **e**, Age-related changes in co-accessibility of loci identified using spatial transcriptomics. *y* axis shows the differences in predicted contact points between young and old hepatocytes (shown as connecting lines in d). Color of the graphs highlight direction of gene expression change as taken from the spatial transcriptomics data between young and old.

**Extended Data Fig. 7:**
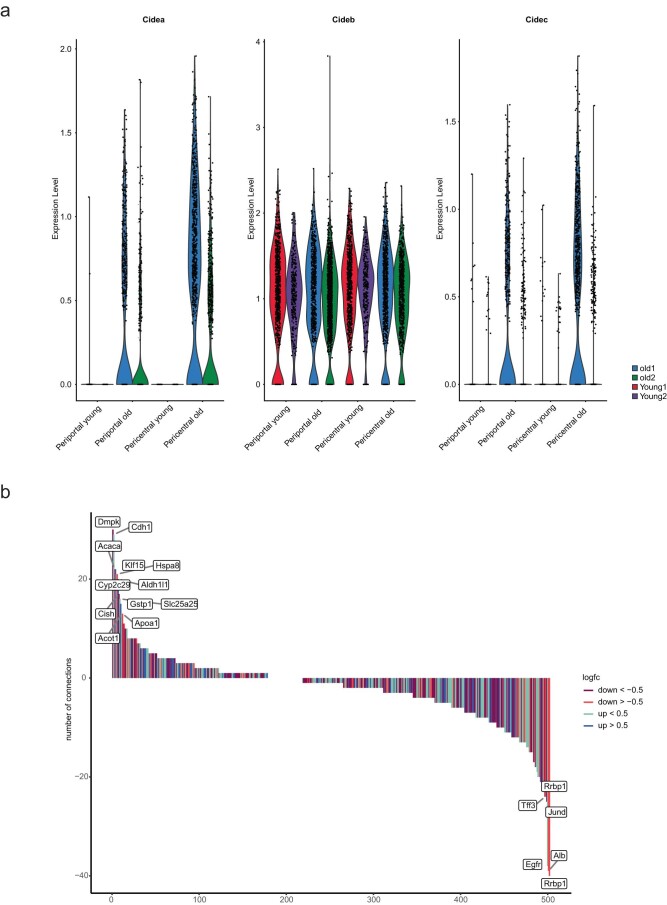
Integration of scATAC with spatial transcriptomics. **a**) Violin plots indicating the expression levels of Cidea, Cideb and Cidec across pericentral and periportal regions in young and old liver - divided by the two biological replicates. **b**) Age-related changes in co-accessibility of loci identified using spatial transcriptomics (as calculated for a second biological scATAC-seq replicate). Y-axis shows the differences in predicted contact points between young and old hepatocytes. Colour of the graphs highlight direction of gene expression change as taken from the spatial transcriptomics data (Supplementary Table 3) between young and old.

How do chromatin alterations connect to the transcriptional program to drive age-related phenotypes? To address this question in more detail, we went back to the differentially expressed and genes that were changed with age and showed a zonated expression. We identified two members of the Cide gene family (Cidea and Cidec, or Fsp27) to be upregulated specifically in old pericentral hepatocytes (Fig. 4a,b and Extended Data Fig. 7a).

Cideb, on the other hand, was expressed across both ages and zones. We used this gene family as a paradigm to understand the connection between chromatin, transcription and phenotype as the expression showed a very clear distribution. In addition, all three Cide proteins have been shown to bind to LDs and to modulate LD dynamics^43–45^. Overexpression of Cide proteins in hepatocytes was sufficient to generate large LDs^45,46^ and using electron microscopy, we found that the mean size of LDs increased 4-fold with age (Fig. 4c), which correlated well with the increased pericentral expression of *Cidea* and *Cidec*. We then turned to our scATAC-seq dataset and probed whether there was an underlying alteration in accessibility at the Cidea locus, potentially explaining the increase in expression and used co-accessibility analysis. Co-accessibility can predict potential interactions between two loci^47^, similar to a Hi-C type approach. Indeed, we observed a specific age-dependent increase in accessibility at the *Cidea* locus (Fig. 4d). In addition, we identified the increased usage of a potential intronic enhancer within *Cidea* as marked by H3K27ac (Fig. 4d), suggestive of stronger enhancer-promoter interactions with age. Given the apparent correlation between locus opening, potential enhancer engagement and transcription output at the *Cidea* locus, we next asked whether changes in co-accessibility might be a good predictor for differential gene expression on a global scale. We used genes differentially expressed between young and old. In addition, we calculated the difference in chromatin co-accessibility for those genes based on the number of predicted interactions (Fig. 4e and Extended Data Fig. 7b). In line with previous reports^48^, we did not detect a general correlation between an increase in co-accessibility and transcription, indicating that co-accessibility is not a determinant for transcription. Taken together, integration of scATAC- with spatial RNA-seq data confirms that alterations in chromatin states are linked to gene expression differences. However, on a global level, we observed a disconnect between chromatin alterations and transcriptional output, suggesting some decoupling of chromatin states and transcription with age. This finding supports recent observations we made on a bulk level in the aging liver^11^.

### Cellular heterogeneity in gene expression increases with age in a zonated and ploidy-dependent manner

**Fig. 5:**
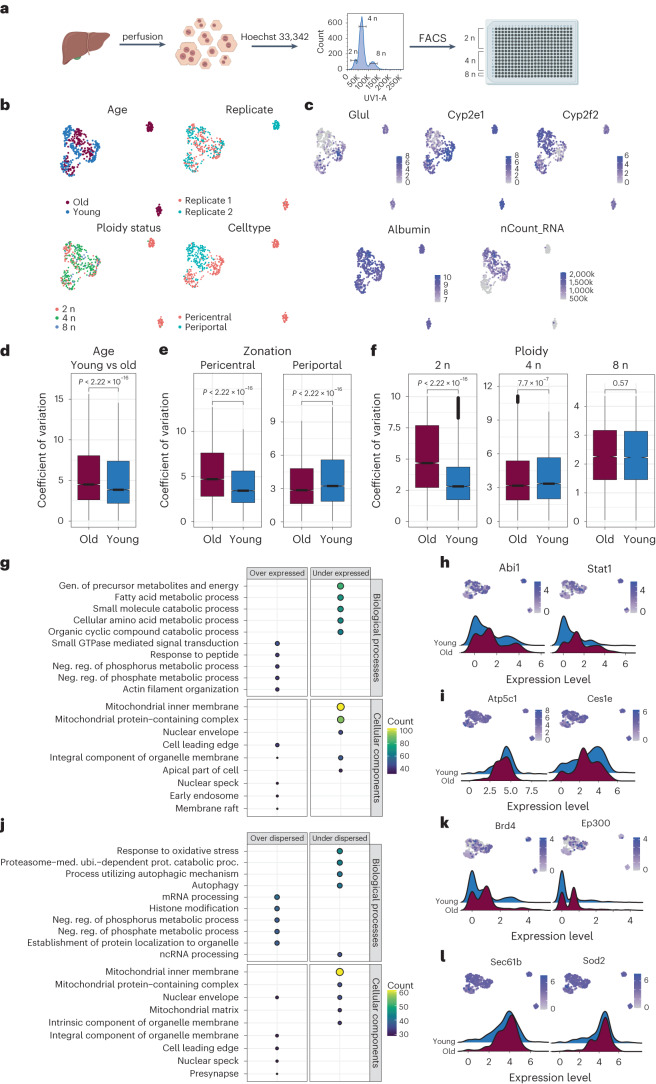
Transcriptional variability in hepatocytes upon aging. **a**, Experimental overview. **b,** Different features of the individual cells projected in a UMAP plot. **c,** Gene expression levels of hepatocyte and zonation markers projected in a UMAP plot. **d–f,** Transcriptional variability upon aging (d), in pericentral and periportal zones (e) and in the differently ploid hepatocytes (f) expressed as coefficient of variation of all detected genes. Significance was calculated using Wilcoxon test within geom_signif function. The lower and upper hinges of the boxplot correspond to the first and third quartiles (25th and 75th percentiles) while the middle line is median and the whiskers extend to 1.5 × interquartile range (IQR) from both lower and upper hinges. The notches extend 1.58 × IQR/sqrt(n), which is roughly 95% confidence intervals (CIs) for comparing medians, **g,** Biological processes (upper panel) and Cellular components (lower panel) for differentially expressed genes. **h**, Examples of overexpressed and **i**) under-expressed genes as feature plot (upper panel) and ridge plot (lower panel). **j**, Biological processes (upper panel) and Cellular components (lower panel) for differentially dispersed genes. Gen.: Generation;Neg reg.: negative regulation;med.: mediated;ubi: ubiquitin;prot: protein;proc.: process. **k**, Examples of over-dispersed and l) under-dispersed genes as feature plot (upper panel) and ridge plot (lower panel).

**Extended Data Fig. 8:**
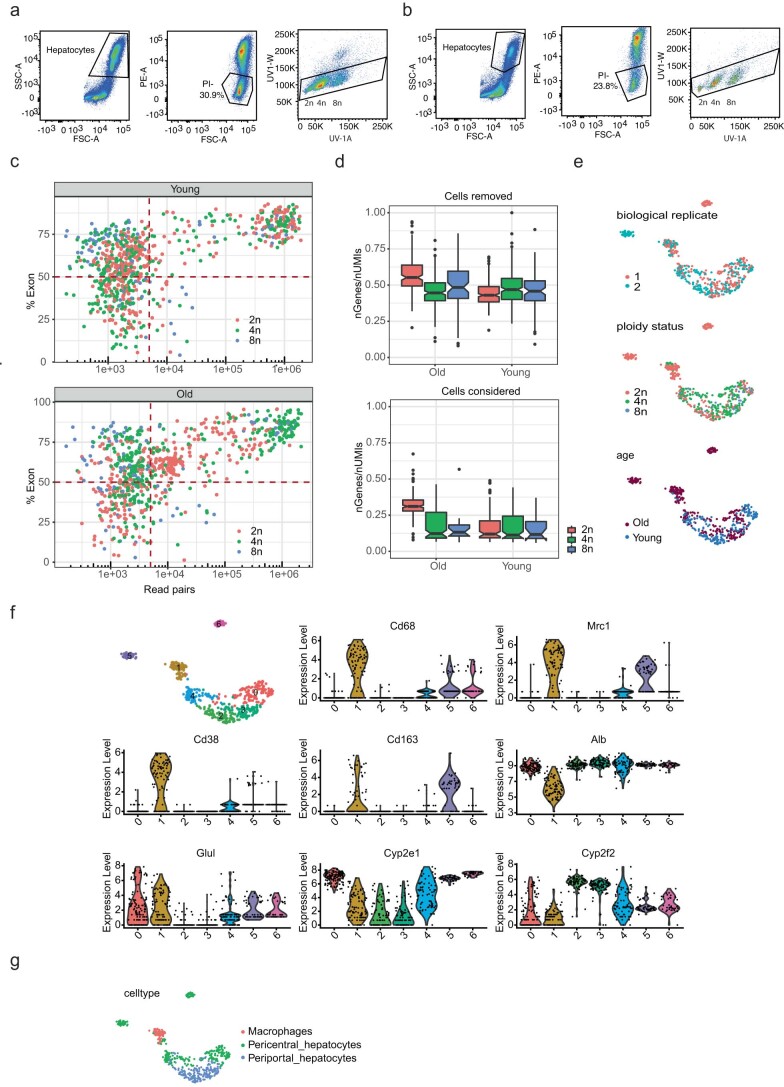
Integration of scATAC with spatial transcriptomics. a,b) Sorting strategy to isolate hepatocytes with different ploidy levels for SMART-seq3 from young (a) and old (b) livers. c) Initial filtering based on number of reads and percentage of exons detected per cell for all sequenced cells. Only cells that fell into the upper right quadrant were taken for further processing. d) Plotting the number of genes/UMI for cells that passed / not passed the initial filter to highlight successful separation of good / bad quality cells using the method described in (c). The lower and upper hinges of the boxplot correspond to the first and third quartiles (25th and 75th percentiles) while the middle line is median and the whiskers extend to 1.5 × interquartile range (IQR) from both lower and upper hinges. The notches extend 1.58 × IQR/sqrt(n), which is roughly 95% confidence intervals (CIs) for comparing medians e) Feature plots representing the described characteristics of the dataset. f) Expression level of hepatic and Kupffer cell markers in the indicated Seurat clusters. g) Based on the expression data, cell type identity was projected onto the individual clusters. The Kupffer cell cluster (cluster #1) was removed from any further analysis, but is still present in the uploaded count matrix available under E-MATB-12579.

Finally, we wanted to address the question of transcriptional variability with age and whether or how it would relate to zonation upon aging. To this end, we generated a high-quality scRNA-seq dataset of hepatocytes using Smart-seq3 from 2 young (3–5 months) and 2 old (18–20 months) male mice. Livers were perfused and viable hepatocytes were FACS-sorted based on size (Fig. 5a and Extended Data Fig. 8a,b). In addition, we recorded ploidy levels of hepatocytes. We performed stringent filtering and initial processing using Seurat^49^ (Extended Data Fig. 8c–e and Supplementary Table 2). We noticed that we purified a population of Kupffer cells (macrophages) specifically in the old livers (Extended Data Fig. 8f,g), which we removed from any subsequent analysis, leaving 545 hepatocytes in total. Projection of the scRNA-seq data in a UMAP revealed three clusters. In the larger cluster, cells were not separated by age or ploidy status (Fig. 5b). This finding is in line with a previous publication that ploidy cannot be distinguished in scRNA-seq data without prior knowledge^50^. However, the data showed a clear separation between pericentral and periportal hepatocytes (Fig. 5b,c). In addition, we observed two smaller, separate clusters containing old diploid hepatocytes, each coming from one old individual (Fig. 5b). These two clusters expressed high levels of Cyp2e1 and albumin as well as low levels of Cyp2f2, suggesting that they consist of pericentral hepatocytes and were characterized by low RNA content (Fig. 5c). Importantly, cells in these two clusters represent the majority (92.70%) of all old diploid pericentral hepatocytes.

**Extended Data Fig. 9:**
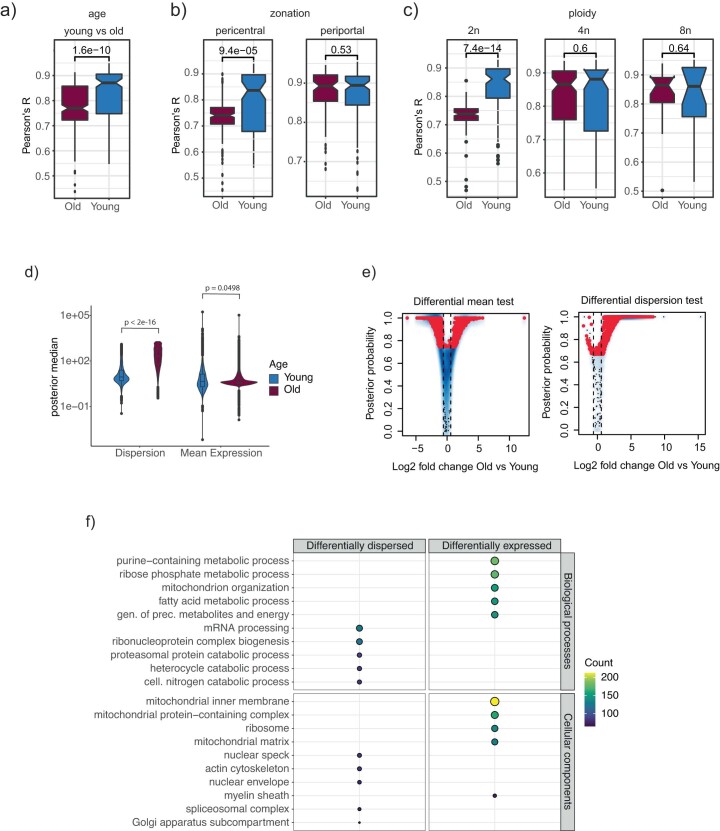
Transcriptional Variability. **a–c**) Transcriptional variability upon ageing (**a**), in pericentral and periportal zones (**b**) and in the differently ploid hepatocytes (**c**) expressed as Pearson Coefficient of all detected genes. **d**) Violin plots for differential dispersed and expressed genes in the Tabula Muris senis dataset. Dispersion and expression was calculated using BASiCS. Significance for the figures a-d was calculated using Wilcoxon test within geom_signif function. The lower and upper hinges of the boxplot correspond to the first and third quartiles (25th and 75th percentiles) while the middle line is median and the whiskers extend to 1.5 × interquartile range (IQR) from both lower and upper hinges. The notches extend 1.58 × IQR/sqrt(n), which is roughly 95% confidence intervals (CIs) for comparing medians **e**) Volcano plot for differentially dispersed genes as found in Tabula Muris senis data of flow sorted liver cells. **f**) Biological processes (upper panel) and Cellular components (lower panel) for differentially expressed and dispersed genes.

After the initial analysis of the scRNA-seq data, we returned to the question of transcriptional variability, which has been described as a consequence of aging. We used the coefficient of variation and cell to cell Pearson’s correlation (Fig. 5d–f and Extended Data Fig. 9a–c) as metrics to compare variability between age, zonal identity and ploidy class. We did observe significant increase in expression variability upon age (Fig. 5d and Extended Data Fig. 9a). Upon dissecting at zonal and ploidy levels it became apparent that the increase in transcriptional variability was largely contributed by the diploid pericentral hepatocytes of old individuals (Fig. 5e,f and Extended Data Fig. 9b,c). Thus, polyploidy levels seem to protect against transcriptional variability in line with previous reports^51^. To identify genes that contributed to the age-dependent increase in noise, we used a regression model implemented within BASiCS^52^. By this means, we were able to compare the differentially expressed and differentially variable genes between young and old cells. The differential test obtained 2,535 (1,570 downregulated and 965 upregulated) and 1,879 (964 down and 915 up) genes significantly differentially expressed and dispersed, respectively (Supplementary Table 5). Strikingly, differentially expressed and dispersed (variable) genes showed a clear functional separation with respect to biological processes affected (Fig. 5g, j). GO enrichment analysis showed that an increase in cell-to-cell variability was associated with genes involved in mRNA processing RNP complex biogenesis, indicating that genes involved in gene expression regulation showed a particular increase in variability with age. On the other hand, differentially expressed genes were enriched for GO terms that deal with metabolic processes, translation and mitochondrial organization. Finally, we carried out the same analysis with the liver tissue from the Tabula Muris senis consortium generated using flow cytometry and Smart-seq2 (FACS data) of male individuals at 3 and 18 months of age.

In the TMS data, we saw a much stronger effect on transcriptional noise (Extended Data Fig. 9d,e), which might be explained by different composition in ploidy levels that were unfortunately not reported in the TMS data. Importantly, we observed a similar impact on biological pathways with respect to differentially expressed and dispersed genes (Extended Data Fig. 9f). This analysis of TMS data is in line with a recent report that showed a general increase in transcriptional variability across various tissues based on this dataset^53^. In summary, we observed an increase in cell-to-cell variability within the transcriptome over age mainly driven by diploid hepatocytes. Interestingly, differentially expressed and dispersed genes belong to different functional categories.

## Discussion

The question of how the direct microenvironment of a cell within a tissue affects the aging trajectory has not been extensively explored. A few studies investigated the role of the microenvironment, particularly on the fate of tissue-resident stem cells, in which age-dependent perturbations of for example the vascular niches trigger the loss of functional hematopoietic stem cells and osteoprogenitors^54^. Indeed, general attrition of vascularization has been recently reported occurring in multiple organs, including the liver^55^ indicating that tissue microenvironments experience profound alterations with age. This is in line with the observation that aging is accompanied by a decline in blood flow in the liver^56^. Given the importance of the vascular system in setting up the division of labor of hepatocytes, the liver represents an ideal tissue to address the consequences of tissue organization and location on one cell type.

Based on spatial transcriptomics, aging is associated with a change in metabolic related terms, particularly amino acid and lipid metabolism as well as mitochondrial energy production.

Functional metabolic seahorse assays show that the mitochondrial function is indeed altered in line with previous reports^57^. Although pericentral hepatocytes were characterized by an increase in SRC, this is strongly diminished in periportal hepatocytes (Fig. 2d). Surprisingly, we noticed a significantly lower expression of E-cadherin, at the protein and gene expression level in our FACS analysis (Fig. 2c). A decreased expression of E-cadherin has been shown to affect HCC progression^58^, linked to periportal fibrosis^59^ and has been associated with decreased mitochondrial membrane potential in tumor microenvironments^60^. Further studies on the role of E-cadherin and other junction molecules in aging liver could potentially give molecular insights into the function of mitochondrial membrane potential in age- related metabolic liver diseases. Next to the insights into the connection of micronenvironmental changes and metabolic as well as epigenomic changes in the aging liver, the data represent a valuable resource for researchers interested in liver organization. Although the scATAC-seq data will allow the interrogation of chromatin states in most liver-resident cell types, the spatial transcriptomics data will mostly give insight into hepatic functions as the hepatocyte are dominating the transcriptional profiles on the spots. However, manual inspection of marker cell types indicates that also the spatial data can be used to interrogate non-parenchymal cells, particularly Kupffer, endothelial and stellate cells (Extended Data Fig. 2a–c). Finally, the scRNA-seq data will allow a detailed dissection of hepatocyte aging at high resolution. One limitation of this study is the fact that we profiled only male mice. Given that there are known difference in aging between the sexes, for instance in drug metabolism^61^, a central activity of the liver, it is plausible that there will be differences in the pathways deregulated at older age.

The most apparent and macroscopic alteration with aging to liver physiology is the accumulation of large LDs in a zonated pattern, with the bulk of LDs being localized in hepatocytes around the central vein of the liver lobule. Using spatial transcriptomics we explored the age-dependent changes that occur within the central to portal axis of the liver lobule. Interestingly, we identified members of the Cide gene family to be predominantly upregulated in the central area of the liver lobule. Cidea, Cideb and Cidec are important regulators of LD dynamic and growth. Indeed, an increase in expression of Cidec has been shown to lead to growth of LDs^62^, suggesting that the increase in Cidea and Cidec expression might be one underlying reason for the increase in LD size with age. The changes in Cidea expression are also encoded in the epigenome. As our scATAC data provided enough resolution to investigate zonation- and age-dependent differences, we could show that the locus encoding for Cidea is remodeled with age and co-accessibility is increased. The presence of H3K27ac indicated that during aging, an intronic enhancer is associated with the pericentral increase of Cidea expression in hepatocytes. Such an increase of expression in Cidea and Cidec has also been linked to the development of hepatic steatosis^63,64^ and prolonged hepatic lipid storage can lead to metabolic dysfunction in the liver, which has been linked to increased cellular senescence and inflammation^65,66^. Ultimately, this development can lead to advanced forms of NAFLD^67^. Thus, it is no surprise that aging is the most common cause for the progression of NAFLD.

All omic technologies showed a clear signature of aging in hepatocytes. However, the observed transcriptional changes were not always in alignment with epigenetic alterations, which was particularly obvious in the case of ATAC and RNA-seq. These results indicated a global decoupling of chromatin and RNA, an observation that we recently reported also on the bulk level^11^. Intriguingly, genes involved in post-transcriptional processing are among the top-dispersed genes, suggesting that this layer of gene expression regulation might be deregulated and more stochastic with age. One part of this layer would be mRNA splicing and indeed, there have been several reports over the last years that the process of splicing is strongly impacted by age and might itself contribute to aging^68–70^. Totally unexplored as of now is the role of mRNA stability and storage with age. The decoupling of chromatin state and transcription is reminiscent of the decoupling of mRNA and protein levels with age^71^. Together, these data suggest that there is a progressive loss of cohesion between the different layers of gene expression that might contribute to the aging process.

## Methods

### Mice

C57BL/6 N male young (3–4 months) and old (18–22 months) mice were bred and maintained in the mouse facility of Max Planck Institute for Biology of Aging following ethical approval by the local authorities (Landesamt für Natur, Umwelt und Verbraucherschutz Nordrhein-Westfalen). The lights were controlled by timers and set a photoperiod of 12 h of light from 6 am until 6 pm (with a 15-min twilight period). The room temperature (RT) was 22 ± 2 °C and the relative humidity 50% ± 5%. All mice were fed with a standard diet ssniff M-Haltung, phyt.-arm (gamma irradiated).

### Immunohistochemistry

Livers were excised postmortem and fixed directly into 4% PFA for 24 h at 4 °C, washed twice with 1× PBS, embedded into paraffin blocks and cut into 5-μm sections. For O-R-O staining and spatial transcriptomics, freshly dissected liver tissues were frozen in Tissue-Tek OCT compound (Sakura) and cut into 7-μm and 10-μm cryosections, respectively.

For IHC stainings, sections of paraffin-embedded samples were deparaffinized by immersion of the slides into the following buffers; 20 min in Xylol, 2 min. 100% EtOH, 2 min. 96% EtOH, 75% EtOH and 1× PBS and washed three times with H_2_O for 5 min each. Endogenous peroxidase was quenched by immersion for 15 min in peroxidase blocking buffer (0.04 M NaCitrate pH 6.0, 0.121 M Na2HPO4, 0.03 M NaN3, 3% H_2_O_2_). After three washes with tap water, slides were subjected to heat-induced epitope retrieval with 10 mM NaCitrate, 0.05% Tween-20, pH 6.0, washed for 5 min with 1× PBS, blocked 60 min with blocking buffer + 160 µl ml^−1^ Avidin D and incubated with primary antibodies diluted (1:200 Plin2) in blocking buffer + 160 µl ml^−1^ biotin overnight at 4 °C. After three 5-min washes with PBST, the samples were incubated with the secondary antibody 1:1000 diluted in blocking buffer for 1 h at room temperature, followed by three 5-min washes with PBST and incubation for 30 min with 1x PBS + 1:60 Avidin D + 1:60 Biotin. After three 5-min washes with PBST the samples were stained with 1 drop of DAB chromogen in 1 ml substrate buffer, washed with 1× PBS and counterstained with hematoxylin for 4 min, washed with tap water and distilled H_2_O and dehydrated 1 min in each buffer; 75% EtOH, 96% EtOH, 100% EtOH and xylol and mounted with Entellan.

### H&E staining

Following deparaffinization, slides with tissues washed with distilled and tapped water and stained with hematoxylin for 5 min, followed by five washes in tapped water and staining with eosin Y for 3 min, followed by three washes with tap water, dehydration and mounting in Entellan.

### O-R-O and Sirius red staining

O-R-O and Sirius red staining were used to visualize neutral lipids and collagen, respectively, and were performed according to standard procedures. O-R-O staining was performed on 7-μm-thick frozen liver sections that were fixed in 4% paraformaldehyde (PFA) for 10 min, followed by staining with 0.3% O-R-O (Sigma) in isopropanol/water (60:40 vol/vol) for 15 min. Sirius red was performed on deparaffinized liver sections that were incubated for 1 h at RT in Picro Sirius red solution (ab150681, Abcam), followed by washes in acetic acid and alcohol solutions.

### RNAscope 2.5 HD Duplex

Liver tissue was placed in a cassette, fixed in 4% PFA dissolved in PBS (pH 7.4) for 24 h at 4°, washed twice with 1X PBS, and embedded into paraffin blocks. Then, 7-μm-thick sections were processed as described below. Detection of *Cyp2f2* (Cat No. 451851), *Alb* (Cat No. 4437691), *Cyp2e1* (Cat No. 402781-C2) and *Glul* (Cat No. 426231-C2) mRNA was performed using a chromogenic in situ hybridization technique (RNAscope 2.5 HD Duplex Assay, Advanced Cell Diagnostics) according to the manufacturer’s instructions. RNAscope 2.5 Duplex positive control probes PPIB-C1 and POLR2A-C2 (Cat No. 321651) were processed in parallel with the target probes. All incubation steps were performed using the ACD HybEz hybridization system (Cat No. 321462). Sections were mounted on SuperFrost Plus Gold slides (Thermo Fisher Scientific), dried at RT, briefly rinsed in autoclaved Millipore water, air-dried, baked at 60 °C for 1 h and deparaffinized. Afterward, slides were treated with hydrogen peroxidase for 10 min. and submerged in Target Retrieval (Cat No. 322000) at 98.5–99.5 °C for 30 min, followed by two brief rinses in autoclaved Millipore water. A hydrophobic barrier was then created around the sections using an ImmEdge hydrophobic barrier pen (Cat No. 310018). Sections were incubated with Protease Plus (Cat No. 322330) for 30 min. The subsequent hybridization, amplification and detection steps were performed according to the manufacturer’s instructions (2.5 HD Duplex Detection kit, Chromogenic, Cat No. 322500). Sections were counterstained with 50% hematoxylin staining and mounted with VectaMount permanent mounting medium (Cat No. H-5000).

### Microscopy

Sirius red, RNAscope and H&E stainings were captured with a Nikon Ti2 Eclipse microscope, equipped with a Nikon Digital Sight D5-VI1 color camera. O-R-O stainings and IHC stainings were taken using a Nikon Eclipse Ci microscope, with a color camera or a S360 Hamamatsu Slidescanner (CECAD Imaging Facility, Cologne). Color brightfield images of HE staining for the spatial transcriptomics experiment were captured with a Nikon Ti2 Eclipse microscope, equipped with a Nikon Digital Sight D5-VI1 color camera and using a 10×/ 0.3 NA CFI Plan-Flour objective. Tilescan image acquisition was carried out using the NIS Elements software.

### Transmission electron microscopy

Livers were excised postmortem, tissue cubes were cut out (1 × 1 × 1 mm) and fixed directly into 2%GA, 2%FA in 0.1 M CaCodylate buffer. Afterwards, samples were rinsed in 0.1 M cacodylate buffer (pH 7.2) and incubated in 1% OsO_4_ and 1% potassium ferrocyanid in 0.1 M cacodylate buffer (pH 7.2) for 3 h at 4 °C.

Liver tissues were dehydrated using ascending ethanol series, transferred to propylene oxide and finally embedded in epoxyresin for 72 h at 62 °C. Ultrathin sections (70 nm) were cut with a diamond knife (Diatome) on an ultramicrotome (EM-UC6, Leica Microsystems) and placed on copper grids. Ultrathin sections were contrasted with 1.5% uranylacetate (Plano GMBH) and lead citrate (Reynolds solution). Images were acquired with a transmission electron microscope (JEOL JEM 2100Plus), camera OneView 4 K 16 bit (Gatan), and software DigitalMicrograph (Gatan) at 80 kV at room temperature.

### Liver perfusion and flow cytometry

Livers were dissociated using the Miltenyi liver perfusion kit (130-128-030) following the manufacturer’s instructions. For sorting pericentral and periportal hepatocytes, the isolated hepatocytes were washed two times with staining buffer (1× PBS, 2 mM EDTA, 0.5% BSA) and 1–7 million hepatocytes were stained with 1:50 FcX, 1:100 PE-anti-E-cadherin, 1:100 APC-anti-CD73 for 1 h at room temperature. Cells were washed two times with staining buffer, cells were filtered through a 100 μm strainer and dead cells were excluded with DAPI. For the sc-RNA sequencing experiment the isolated hepatocytes were washed two times with staining buffer (1x PBS, 2 mM EDTA, 0.5%BSA) and 1 million hepatocytes were stained with Hoechst (15 μg ml^−1^) and Reserpine (5 μM) for 30 min at 37 °C. Dead cells were excluded with PI staining (1 μg ml^−1^). Cells were sorted using a BD FACSARIA IIIU or Fusion Cytometer and 130um nozzle. The data were analyzed using the BD FACSDiva and FlowJo software.

### Mitochondrial function measurement

Mitochondrial function was evaluated by measuring the oxygen consumption rate with the Seahorse XFe96 Extracellular Flux Analyzer (Agilent). XFe96 cell culture plates were coated with Collagen-I (40 μg ml^−1^) overnight at 4 °C and then washed 2x with 1X DPBS before 6,000 murine primary hepatocytes were seeded onto each well. Cells were cultured overnight in DMEM + GlutaMAX containing 10% FBS and 1x PenStrep under humidified conditions at 37 °C with 5% CO_2_. Cells were washed 2x with assay media composed of XF DMEM medium (pH 7.4) supplemented with glucose (10 mM), pyruvate (1 mM) and glutamine (2 mM). Cells were cultured in assay media and incubated for 1 h at 37 °C in a non-CO_2_ incubator. The Seahorse XF Mito Stress test was used to measure the oxygen consumption rate response after the sequential injection of oligomycin (1.0 μM), FCCP (1.0 μM) and Rot/AA (0.5 μM), according to the manufacturer’s instructions. The data were normalized to cell confluency. Brightfield images were acquired with the EVOS FL Auto 2 system (Thermo Fisher Scientific) using a 4×/ 0.13 NA Plan Fluorite objective. After calibrating the system for the Seahorse multiwell plates, tile scan images of whole wells could be automatically captured. Confluency was analyzed by training a supervised machine learning network for automatic pixel classification using Ilastik^72^. After classification, subsequent analysis was carried out in FIJI^73^. Therefore, the label masks were cleaned up by two rounds of the binary operation ‘open’ using the Biovoxxel toolbox plugin (10.5281/zenodo.5986130), and the covered areas in each well could be measured and normalized to the corresponding well area to get the final percent of confluency.

### Genomic DNA extraction and qPCR for mitochondrial content

Liver genomic DNA was extracted using the NucleoSpin Tissue XS, Micro kit for DNA (Macherey and Nagel, 740901.50). Real-time PCR was performed with primers specific to the cyto-b mitochondrial locus (forward: 5′-TCCGATATATACACGCAAACG-3′, reverse: 5′-ATAAGCCTCGTCCGACATGA-3′) and results were normalized to total genomic DNA using primers for actin promoter locus (forward: 5′-TGCCCCATTCAATGTCTCGG-3′, reverse: 5′-ATCCACGTGACATCCACACC-3′).

### mRNA extraction and qPCR for Cyp2f2 and Glul expression

To verify the relative abundance of expression of the respective markers of the sorted cells, CD73^+^ pericentral and E-cadherin^+^ periportal cells were isolated with flow cytometry (see methods above) from 4 individual (2 young and 2 old) mice and mRNA was extracted with the Dynabeads mRNA DIRECT Purification Kit (61011 Thermo Fisher Scientific). Reverse transcription was performed with the Maxima H Minus Reverse Transkriptase (EP 0751 Thermo Fisher Scientific) and the cDNA was used for qPCR with primers for Cyp2f2 (forward: 5′-CTTCCTGATACCCAAGGGCAC-3′, reverse: 5′-CTGAGGCGTCTTGAACTGGT-3′) and Glul (forward: 5′-CCACCGCTCTGAACACCTT-3′, reverse: 5′-TGGCTTGGACTTTCTCACCC-3′). The results were normalized to Actin expression (forward: 5′-ACCGGTGCAGAGACATTGGAGTTCAAC-3′, reverse: 5′-GTCGACTCAGATCCCGAGGCAGAGTC-3′).

### Lipidomics

#### Lipid extraction from liver tissue

For the lipidomic analysis of liver tissue, 20 mg of snap-frozen tissue samples were homogenized using precooled (liquid N_2_) metal balls (5 mm diameter) in a Qiagen Tissue Lyser for 1 min at 25 Hz. The pulverized tissue was resuspended in 1 ml pre-cooled (−20 °C) extraction buffer (MTBE (methyl tert-butyl):MeOH 75:25 [v:v]), containing 0.2 µl EquiSplash Lipidomix as an internal standard. The resuspended samples were homogenized for additional 5 min at 15 Hz in the TissueLyser.

After efficient tissue lysis, the samples were incubated for additional for 30 min on a thermomixer at 1,500 rpm and at 4 °C. To remove precipitated material from the samples, the Metal balls were removed and all samples were centrifuged for 10 min at 4 °C and 21.000 *g*. The supernatants were transferred to a new tube and 500 µl H_2_O:methanol 3:1 [v:v] were added before further incubating the extracts for additional 10 min at 1,500 rpm and 15 °C on a thermomixer. After this final incubation step the polar and lipid phases were separated in a 10 min centrifugation step at 16,000 *g* and 15 °C. The upper phase, MTBE-phase was transferred to a new tube and stored with the obtained insoluble pellets at −80 °C for lipidomic analysis and protein extraction and quantification (BCA).

#### Liquid chromatography high-resolution mass spectrometry-based analysis of lipids

The stored (−80 °C) lipid extracts were dried in a SpeedVac concentrator before analysis and lipid pellets were resuspended in 200 µl of a UPLC-grade acetonitrile: isopropanol (70:30 [v:v]) mixture. Samples were vortexed for 10 s and incubated for 10 min on a thermomixer at 4 °C. Resuspended samples were centrifuged for 5 min at 10,000 *g* and 4 °C, before transferring the cleared supernatant to 2 ml glass vials with 200 µl glass inserts. All samples were placed in an Acquity iClass UPLC sample manager at 6 °C. The UPLC was connected to a Tribrid Orbitrap HRMS, equipped with a heated electrospray ionization ion source (ID-X, Thermo Fischer Scientific).

Of each lipid sample, 1 µl was injected onto a 100 ×2.1 mm BEH C_8_ UPLC column, packed with 1.7 µm particles. The flow rate of the UPLC was set to 400 µl/min and the buffer system consisted of buffer A (10 mM ammonium acetate, 0.1% acetic acid in UPLC-grade water) and buffer B (10 mM ammonium acetate, 0.1% acetic acid in UPLC-grade acetonitrile/isopropanol 7:3 [v/v]). The UPLC gradient was as follows: 0–1 min 45% A, 1–4 min 45–25% A, 4–12 min 25–11% A, 12–15 min 11-1% A, 15-18 min 1% A, 20–18.1 min 1–45% A and 18.1–22 min re-equilibrating at 45% A. This leads to a total runtime of 22 min per sample.

The ID-X mass spectrometer was operating either for the first injection in positive ionization mode or for the second injection in negative ionization mode. In both cases, the analyzed mass range was between m/z 160–1,600. The resolution (R) was set to 120.000, leading to approximately 4 scans per second. The RF lens was set to 60%, while the AGC target was set to 250%. The maximal ion time was set to 100 ms and the heated electrospray ionization source was operating with a spray voltage of 3.5 kV in positive ionization mode, while 3.2 kV were applied in negative ionization mode. The ion tube transfer capillary temperature was 300 °C, the sheath gas flow 60 arbitrary units (AU), the auxiliary gas flow 20 AU and the sweep gas flow was set to 1 AU at 340 °C.

All samples were measured in a randomized run order, and targeted data analysis was performed using the quan module of the TraceFinder 4.1 software (Thermo Fischer Scientific) in combination with a sample-specific in-house-generated compound database. Peak areas of each peak were normalized to the internal standards from the extraction buffer and to the fresh weight of the tissue.

### Spatial transcriptomics

#### Tissue and library preparation

Liver specimen from 2 young and 2 old mice were cryopreserved and sections of 8 mm × 8 mm × 10μm specimens. Libraries were prepared using the Visium Spatial Gene Expression solution from 10x Genomics using 30 min permeabilization time. Libraries were prepared according to the manufacturer’s instruction and sequenced on an Illumina NovaSeq 6000. Sequencing data was initially quality controlled and preprocessed using the 10x Genomics SpaceRanger framework (V. 1.2.2).

#### Dimensionality reduction and individual analysis of datasets

Young and old liver tissue slides were analyzed individually in R (V. 4.0.0) using the Seurat package (V. 4.0.4) (ref. ^49^). Count matrices were normalized and scaled using the *SCTransform* function with standard parameters. Relative gene expression visualization of known hepatic pericentral and periportal marker genes on the spots of the tissue slides was performed with the *SpatialFeaturePlot* function.

#### Dataset integration

To assess batch effects between tissue slides, we merged the processed slides using the *merge* function and normalized and scaled without any further batch correction. Principal component analysis for was performed on the 2,000 most variable features. The top 50 genes associated with the first principal PCA component were visualized with the *VizDimLoadings* functions and intersected with the hepatocyte specific aging genes list from^17^. Integration of young and old liver tissue slides was performed in a stepwise manner as an integration of all datasets together would remove all potential differences between young and old datasets. First, the preprocessed young and old tissue slide datasets were integrated separately per age group using canonical correlation analysis described in^16^. Second, both combined datasets were merged and filtered for spots to have at least 1000 and at most 7000 genes expressed. Subsequently, the joined count matrix was scaled and normalized together using the *NormalizeData* and *ScaleData* function.

#### Dimensionality reduction of integrated datasets

We performed PCA on the preprocessed data (*RunPCA* function). The first 10 principal components covered most of the dataset’s variance and were considered a good approximation to the data as assessed by an elbowplot (*Elbowplot* function). The first 10 principal components, therefore, served as input to UMAP for further dimension reduction and visualization. Known canonical liver zonation marker genes were visualized with the *Featureplot* function.

#### Differential expression testing between young and old liver tissue slides

Differential expression testing was done by using the *FindMarkers* function. Genes had to show at least an average log_2_-fold change of ±0.25 to be considered for testing. Testing was performed using the *MAST* library (V. 1.19.0) (ref. ^18^). Bonferroni correction was applied for multiple testing adjustments of *P* values and a significance threshold of 0.05 was used for all analyses.

### Cytoscape

The Cytoscape (V3.9.1) (ref. ^20^) app iRegulon (V. 1.3) (ref. ^21^) was used to calculate transcription factor predictions. Differentially expressed genes in old (Supplementary Table 3) were used as input for all analysis. iRegulon was run using Mus musculus MGI symbols using the following motif collection: 10k (9712PWMs). Putative regulatory regions as well as motif ranking database were set as 20 kb centered on the transcription start site. Normalized enrichment scores for all transcription factors reported were > 4.

### Liver tissue preparation for scATAC-seq

Liver nuclei (*n* = 4 per experiment/age group) were prepared from frozen tissue specimens by crushing and dounce homogenizing the tissue in 1 ml EZ-buffer (SIGMA) (20 strokes with loose and a tight pestle, respectively) and spun 5 min at 300 *g*. The pellet was incubated on ice for 20 min in EZ-buffer supplemented with DNAseI NEB M0303S (4 U ml^−1^) and 1X DNAseI buffer. Equal volume of EZ-buffer was added and samples were spun 5 min at 500 g and incubated again 10 min on ice in EZ-buffer supplemented with DNAseI NEB M0303S (8 U ml^−1^) and 1X DNAseI buffer. Equal volume of EZ-buffer was added, and samples were spun 5 min at 500 *g*, resuspended in NSB (1087.5 µl 1× PBS, 5.5 µl 2% BSA, 1.5 µl RNase Inhibitor) and filtered three times through a 0.22-µm strainer.

For scATAC-seq, 100,000 (25,000 from each individual biological replicate) nuclei were pooled and resuspended in 50 µl tagmentation mix (10X Genomics)).

For ploidy FACS analysis the nuclei were resuspended in staining buffer and stained for 15 min with PI (1 μg ml^−1^) at RT, before running them in BD LSRFortessa flow cytometer. Analysis was performed with BD FACSDiva and FlowJo softwares.

### scATAC-seq library preparation and sequencing

scATAC-seq targeting 4,000 cells per sample and per dataset was performed using a Chromium Single Cell ATAC Library and Gel Bead kit (10x Genomics, 1000110) according to the manufacturer’s instructions. Libraries were then pooled and loaded on an Illumina NovaSeq sequencer and sequenced to 21,557 median high-quality fragments per cell in the first biological replicate and to 27,028 in the second. Sequencing data were initially quality controlled and preprocessed using the 10x Genomics CellRanger framework (V. 2.1.0).

### scATAC-seq analysis of young and old liver tissue

Region accessibility count data were analyzed using the *cisTopic* library (V. 0 3.0) (ref. ^34^). Cells without any accessible regions were removed and we profiled a total of 6,579 nuclei (4,320/2,259) prepared from young liver tissue and 5,750 nuclei (3,260/2,490) from old liver tissue. We included 166,813/161,102 regions into our analysis that were accessible in at least one cell. The latent Dirichlet allocation model was learned by the *runWarpLDAModels* function for topic numbers ranging from 2 to 50 topics. An appropriate number of topics for our data was selected using the *selectModel* function. This was the case for 32/30 topics, and all downstream analyses use the LDA model learned for 32/30 topics. Nonlinear dimensionality reduction by UMAP was performed by applying the built-in *runUmap* function in cisTopic to the topic-distributions of all cells. Topic-defining regions were derived via the *getRegionsScores*- and *binarizecisTopics*-function. GO term and transcription factor motif analysis of the topic-defining regions was done using *rGREAT* (V. 1.26.0) and *RcisTarget* (V. 1.14)^38^. Transcription factor motifs shown in Fig. 3g were downloaded from the *JASPAR* database (http://jaspar.genereg.net).

To check the robustness of the cisTopic results, we performed a complementary analysis of the same data with *Signac* (V. 1.8.) (ref. ^33^). The cell region count matrix was normalized using the term frequency-inverse document frequency (TF-IDF) normalization method from the *Signac* library (*RunTFIDF*). Initial linear dimensionality reduction was performed with singular value decomposition (*RunSVD*). As recorded in the Signac workflow, the first component of the singular value decomposition was excluded from all downstream analyses as it was highly correlated with the sequencing depth. Nonlinear dimensionality reduction (UMAP) for Extended Data Fig. 3 was generated via the *RunUMAP* function. The dimensions 2 to 30 were used as input for the algorithm.

#### Differential accessibility testing

We used the *FindMarkers* function in the logistic regression framework of^74^ to test for regions that were differentially accessible between young and old hepatocytes, respectively, between periportal and pericentral hepatocytes. We considered only regions detected in at least 5% of the cells for testing. P-values were Bonferroni adjusted to account for multiple testing. If not otherwise stated a significance threshold of 0.05 was used for all analyses.

#### Cell type annotation

Our cell type annotation is based on the imputed gene activity of known liver cell marker genes from *CellAtlas*^75^. To calculate the imputed gene activities, fragments mapping to gene bodies or promoter regions of genes (up to 2 kb upstream of a gene) were summed up using the *GeneActivity* function and subsequently normalized via the *NormalizeData* function from Signac. Periportal and pericentral cell populations were annotated based on the gene activity of *Cyp2e1* and *Cyp2f2* genes.

#### Construction of *cis*-regulatory networks

Co-accessibility scores for the interaction network of the *Cidea* locus were predicted with the *Cicero* library (V. 1.3.8) (ref. ^47^). Reduced dimension coordinates of cells were based on the UMAP projection from *cisTopic*. Connections of co-accessible loci were inferred for young and old hepatocytes separately.

### Bulk RNA-seq data processing and analysis

The TMS bulk RNA-seq data were analyzed by directly using the count matrix provided (10.6084/m9.figshare.8286230.v1). We only used the data from male mice of the age 3 and 18 months. First, genes were filtered using the ‘filterByExpr’ function of edgeR (3.28.1) (ref. ^76^) with the min.count = 3. The differential gene expression analysis between young (3 months) and old (18 months) was carried out using DEseq2 (1.26.0) (ref. ^77^) at the adjusted *P* value of 0.1. Obtained sets of genes were further used for GO Biological Processes enrichment using the ‘enrichGO’ function from clusterProfiler (3.14.3) R package^78^. To remove the redundancy of enriched terms, we used the ‘simplify’ function from clusterProfiler with the default parameters.

### scRNA-seq library preparation and sequencing

scRNA-seq libraries were generated using an early version of the Smart-seq3xpress protocol^79^. Reaction conditions were identical to the published final protocol deposited on protocols.io (https://www.protocols.io/view/smart-seq3xpress-yxmvmk1yng3p/v2). Briefly, cells were sorted into 300 nl lysis buffer containing 0.5 μM oligo-dT primer overlayed with 3 μl Silicon Oil (100 cSt; Sigma-Aldrich). Next, reverse transcription was carried out in 400 nl using 0.75 μM Smart-seq3 TSO. Preamplification of cDNA was performed in 1 μl using SeqAmp polymerase (Takara Bio) for 15 cycles. Subsequently, amplified cDNA was diluted with 9 μl H2O and we used 1 μl for downstream tagmentation using 0.002 μl TDE1 enzyme per cell. Final, indexed libraries were pooled per 384-well plate, cleaned and equimolarly pooled before sequencing. For high-throughput sequencing, linear dsDNA library pool was converted to circular ssDNA using the MGIEasy Library Conversion Kit (App-A; MGI Tech). Next, we used 60 fmol ssCircDNA for generation of DNA nanoballs (DNBs). DNBs were loaded onto a lane of a FCL flow-cell and sequenced PE100 on the G400RS platform (MGI Tech).

### scRNA-seq data processing and analysis

We processed raw fastq reads using zUMIs (V. 2.9.7)^80^ to obtain raw count tables. Within zUMIs, the data was mapped to the mouse genome mm10 using Ensembl annotation version 99 using STAR (V. 2.7.1a). The count matrix was further filtered for genes expressed in at least 3 cells, cells containing minimum 200 genes and 1000 counts. The filtered count matrix was processed using Seurat (V. 4.1.1)^49^ with default parameters as per suggested pipeline using ‘SCTransform’, ‘RunPCA’, ‘RunUMAP’, ‘FindNeighbors’ and ‘FindClusters’ functions. The feature and PCA/UMAP plots generated in this manuscript are through Seurat plotting functions. Cell type annotation was performed using known marker genes for hepatocytes and macrophages. Periportal and pericentral cell populations were annotated based on the expression of *Cyp2e1* and *Cyp2f2* genes. The differential expression analysis for the distinct old diploid pericentral hepatocytes against other hepatocytes was performed using the ‘FindMarkers’ function within Seurat and further filtered for absolute log2FC of 1.5 and adjusted *P* value below 0.05.

#### Preliminary processing of TMS data

We downloaded metadata and raw count tables from Tabula Muris Senis consortium for liver FACS and droplets methods. The TMS FACS and droplets data was filtered for genes expressed in at least three cells, cells containing minimum 250 genes and 2,500 counts for droplets whereas 500 genes and 5,000 UMIs for the FACS data. The filtered count matrix was processed using Seurat (4.1.4)^49^ with default parameters as per suggested pipeline using ‘SCTransform’, ‘RunPCA’, ‘RunUMAP’, ‘FindNeighbors’ and ‘FindClusters’ functions. The feature and PCA/UMAP plots generated in this manuscript are through Seurat plotting functions.

#### Differential expression and dispersion analysis

The differential analysis was performed using the BASiCS (V. 2.8.0) package^52^. Posterior estimates were computed using a Markov chain Monte Carlo (MCMC) simulation with 20,000 iterations and burn-in period 10,000 with a regression model. We used BASiCS to detect differentially expressed and differentially variable genes between old and young hepatocytes. For changes in mean expression between ages, we use the ‘BASiCS_TestDE’ function with EFDR cutoff 0.1. Only genes with no change in mean expression were considered for interpreting changes in variability. We filtered genes with the detection rate of 0.05 in each age and mean overall expression of 1.

Obtained sets of genes from each differentially expressed and variability were further subjected to GO Biological Processes and Cellular Components enrichment analysis using the ‘enrichGO’ function from clusterProfiler (V. 3.14.3) R package^78^. To remove the redundancy of enriched terms, we used the ‘simplify’ function from clusterProfiler (V. 3.14.3) with the default parameters. The pathway enrichment was performed using the ‘enrichPathway’ function from the ReactomePA R package (V. 1.36.0) (ref. ^81^).

### Statistics and reproducibility

Sample sizes were chosen based on previously reported publications (lipidomics, scATAC, metabolic assays)^27,79,82^. The only pre-established exclusion criterion was for replicates that were found to be technically flawed or determined by statistical tests to contain a legitimate outlier data point. When any of the above occurred, the entire replicate was omitted and the whole experiment was repeated, when feasible. Taking into consideration the above exclusion criterion, all experiments were successfully reproduced at least twice (RNAScope and IHC staining (representative images and sections shown), scRNA-seq and scATAC-seq), whereas all other experiments were performed using at least three independent biological replicates. Experimental groups were based on age. Data collection and analysis were not performed blind to the conditions of the experiments.

### Reporting summary

Further information on research design is available in the Nature Portfolio Reporting Summary linked to this article.

### Supplementary information

Reporting Summary

Supplementary Table 1 RNA-seq analysis bulk data.

Supplementary Table 2 Sequencing metrics.

Supplementary Table 3 Differentially expressed genes from spatial transcriptomics (between age and zones).

Supplementary Table 4 Lipidomics results.

Supplementary Table 5 SMARTseq3-express; differentially expressed genes.

### Source data

Source Data Fig. 2 Statistical source data.

## Data Availability

All sequencing data generated for this study is available at Array Express with following accession numbers: spatial transcriptomics from young and old liver: E-MTAB-12809. scATAC-seq of young and old livers: E-MTAB-12706 and E-MTAB-12560; and SMART-seq3xpress data on young and old hepatocytes: E-MTAB-12579. H3K27ac for young and old mice was downloaded from BioProject PRJNA281127. Tabula Muris senis single cell data are available at Gene Expression Omnibus GSE149590. Tabula Muris senis bulk RNA-seq data are available at Gene Expression Omnibus GSE132040 (ref. ^9^). All other data will be provided by the corresponding author upon reasonable request.

## Code availability

All scripts used to analyze data are available on Github: https://github.com/ptessarz/aging_liver.
