# Supplementary material for: Spatial and single-cell profiling of the metabolome, transcriptome and epigenome of the aging mouse liver
Source: Nat Aging. 2023 Nov 9;3(11):1430–45. doi: 10.1038/s43587-023-00513-y (PMC10645594; doi:10.1038/s43587-023-00513-y)
Supplement: Supplementary file 1 — Reporting Summary [file 43587_2023_513_MOESM1_ESM.pdf]

nature portfolio

Corresponding author(s): Achim Tresch & Peter Tessarz

Last updated by author(s): Sep 5, 2023

Reporting Summary

Nature Portfolio wishes to improve the reproducibility of the work that we publish. This form provides structure for consistency and transparency in reporting. For further information on Nature Portfolio policies, see our Editorial Policies and the Editorial Policy Checklist.

Statistics

For all statistical analyses, confirm that the following items are present in the figure legend, table legend, main text, or Methods section.

n/a	Confirmed	
☐	☒	The exact sample size (*n*) for each experimental group/condition, given as a discrete number and unit of measurement
☐	☒	A statement on whether measurements were taken from distinct samples or whether the same sample was measured repeatedly
☐	☒	The statistical test(s) used AND whether they are one- or two-sided<br>*Only common tests should be described solely by name; describe more complex techniques in the Methods section.*
☐	☒	A description of all covariates tested
☐	☒	A description of any assumptions or corrections, such as tests of normality and adjustment for multiple comparisons
☐	☒	A full description of the statistical parameters including central tendency (e.g. means) or other basic estimates (e.g. regression coefficient) AND variation (e.g. standard deviation) or associated estimates of uncertainty (e.g. confidence intervals)
☐	☒	For null hypothesis testing, the test statistic (e.g. *F*, *t*, *r*) with confidence intervals, effect sizes, degrees of freedom and *P* value noted<br>*Give P values as exact values whenever suitable.*
☐	☒	For Bayesian analysis, information on the choice of priors and Markov chain Monte Carlo settings
☒	☐	For hierarchical and complex designs, identification of the appropriate level for tests and full reporting of outcomes
☐	☒	Estimates of effect sizes (e.g. Cohen's *d*, Pearson's *r*), indicating how they were calculated

Our web collection on statistics for biologists contains articles on many of the points above.

Software and code

Policy information about availability of computer code

Data collection	scRNA-seq of liver tissue data was downloaded from Tabula Muris Senis consortium. All the other datasets were generated in-house and the detailed description is provided in the corresponding materials and methods section in the manuscript.
Data analysis	The data analysis was performed mainly using available open source R packages, open source tools and data wrangling was done using custom R scripts. The description of all the parameters used for analysis are provided in detail in the corresponding methods section.

For manuscripts utilizing custom algorithms or software that are central to the research but not yet described in published literature, software must be made available to editors and reviewers. We strongly encourage code deposition in a community repository (e.g. GitHub). See the Nature Portfolio guidelines for submitting code & software for further information.

Data

Policy information about availability of data

All manuscripts must include a data availability statement. This statement should provide the following information, where applicable:

- Accession codes, unique identifiers, or web links for publicly available datasets
- A description of any restrictions on data availability
- For clinical datasets or third party data, please ensure that the statement adheres to our policy

Spatial Transcriptomics from young and old liver: E-MATB-12809 (https://www.ebi.ac.uk/fg/annotare/edit/13596/)
scATAC-seq of young and old livers: E-MATB-12706 (https://www.ebi.ac.uk/fg/annotare/edit/13595/) and E-MATB-12560 (https://www.ebi.ac.uk/fg/annotare/edit/16069/)
SMART-seq3xpress data on young and old hepatocytes: E-MATB-12579 (https://www.ebi.ac.uk/fg/annotare/edit/15744/)

Field-specific reporting

Please select the one below that is the best fit for your research. If you are not sure, read the appropriate sections before making your selection.

☒ Life sciences ☐ Behavioural & social sciences ☐ Ecological, evolutionary & environmental sciences

For a reference copy of the document with all sections, see nature.com/documents/nr-reporting-summary-flat.pdf

Life sciences study design

All studies must disclose on these points even when the disclosure is negative.

Sample size	Sample sizes were chosen based on previously reported publications (lipidomics, scATAC, metabolic assays), such as Cehn et al., JCI insight 2020; Gajendiran et al. L. Cell. Mol. Med. 2018 and Chen et al., Cell 2020
Data exclusions	The only pre-established exclusion criterion was for replicates that were found to be technically flawed or determined by statistical tests to contain a legitimate outlier data point. When any of the above occurred, the entire replicate was omitted and the whole experiment was repeated, when feasible. The number of final replicates used for each analysis is extensively described in the next section.
Replication	Taking into consideration the above exclusion criterion, all experiments were successfully reproduced at least twice. Final number of biological replicates is clearly indicated in each figure legend.
Randomization	Experimental groups were based on age.
Blinding	None of our experiments were blinded, since results were inherently objective and unbiased. Results were subjected to binary interpretation, based on statistical tests. Furthermore, analyses could not be blinded because experiments were performed and analyzed by the same researchers.

Reporting for specific materials, systems and methods

We require information from authors about some types of materials, experimental systems and methods used in many studies. Here, indicate whether each material, system or method listed is relevant to your study. If you are not sure if a list item applies to your research, read the appropriate section before selecting a response.

Materials & experimental systems

n/a	Involved in the study
☐	☒ Antibodies
☒	☐ Eukaryotic cell lines
☒	☐ Palaeontology and archaeology
☐	☒ Animals and other organisms
☒	☐ Human research participants
☒	☐ Clinical data
☒	☐ Dual use research of concern

Methods

n/a	Involved in the study
☒	☐ ChIP-seq
☐	☒ Flow cytometry
☒	☐ MRI-based neuroimaging

Antibodies

Antibodies used	E-cadherin-PE (Biolegend, 147304; LOT B335439; 1:100)<br>CD73-APC (Biolegend; 127210; LOT B291453; 1:100)<br>Plin2 (NOVUS; NB110-40877SS; LOT G-4; 1:200)<br>CD68 (DAKO; PG-M1, M087601-2, 1:200)<br>CD68 (abcam; ab76308, 1:100)<br>MARCO (abcam; EPR22944-64, ab239369, 1:100)<br>Anti-Rabbit Biotin (Perkin Elmer , NEF813001 , goat , 1:1000 (stock 0.5mg/ml in 50% glycerol)<br>Anti-mouse Biotin (Biozol , BA-9200 , goat , 1:1000)
Validation	CD73-APC species reactivity: mouse; tested applications: FC; product citations: 12; https://www.biolegend.com/en-us/products/apc-anti-mouse-cd73-antibody-7893?GroupID=BLG10609<br>E-cadherin-PE species reactivity: human, mouse; tested applications: FC; product citations: 9; https://www.biolegend.com/en-us/products/pe-anti-mouse-human-cd324-e-cadherin-antibody-9276<br>Plin2 species reactivity: human, mouse, rat, bovine; tested applications: WB, Western, IHC, ICC/IF, WHC-P, Dual ISH-IHC; product citations: 33; https://www.novusbio.com/products/perilipin-2-adfp-antibody_nb110-40877#reviews-publications<br>CD68 clone PG-M1 reactivity: human, tested applications: IHC; https://www.agilent.com/en/product/immunohistochemistry/antibodies-controls/primary-antibodies/cd68-%28concentrate%29-76550#productdetails<br>CD68 clone EPR1392Y reactivity: human, mouse, rat, tested applications: IHC, WB, ICC-IF, citations: 10, https://www.citeab.com/

antibodies/721313-ab76308-anti-cd68-antibody-epr1392y
MARCO species reactivity: mouse ; tested applications: IP, IHC-Fr, IHC-P, ICC/IF, WB; https://www.abcam.com/products/primary-antibodies/marco-antibody-epr22944-64-ab239369.html
Anti-Rabbit Biotin (https://www.perkinelmer.com/product/anti-rabbit-igg-biotin-labeled-goat-nef813001ea)
Anti-mouse Biotin (https://www.biozol.de/en/product/vec-ba-9200)

Animals and other organisms

Policy information about studies involving animals; ARRIVE guidelines recommended for reporting animal research

Laboratory animals	C57BL/6N wild type male mice were used in this study<br>Mice were 3-4 months old (young cohort) and 18-22 months old (old cohort). Information about the housing conditions of the mice is provided in the respective section of the manuscript.
Wild animals	The study did not involve wild animals.
Field-collected samples	The study did not involve field-collected samples.
Ethics oversight	Landesamt für Natur, Umwelt und Verbraucherschutz Nordrhein-Westfalen (LANUV NRW) approved the protocols used in this paper.

Note that full information on the approval of the study protocol must also be provided in the manuscript.

Flow Cytometry

Plots

Confirm that:

☒ The axis labels state the marker and fluorochrome used (e.g. CD4-FITC).

☒ The axis scales are clearly visible. Include numbers along axes only for bottom left plot of group (a 'group' is an analysis of identical markers).

☒ All plots are contour plots with outliers or pseudocolor plots.

☒ A numerical value for number of cells or percentage (with statistics) is provided.

Methodology

Sample preparation	The isolated hepatocytes were washed two times with staining buffer (1x PBS, 2mM EDTA, 0.5%BSA) and 1-7million hepatocytes were stained with 1:50 FcX, 1:100 PE-anti-E-cadherin, 1:100 APC-anti-CD73 for 1hr at room temperature. Cells were washed two times with staining buffer and DAPI was added. The hepatocytes were filtered through a 100um strainer before sorting.<br>For the sc557 RNA sequencing experiment the isolated hepatocytes were washed two times with staining<br>558 buffer (1x PBS, 2mM EDTA, 0.5%BSA) and 1 million hepatocytes were stained with Hoechst<br>559 (15µg/ml) and Reserpine (5uM) for 30min at 37°C. Dead cells were excluded with PI staining<br>560 (1µg/ml).
Instrument	For the cell sorting we used the BD FACSAria IIIu and Fusion instruments with a 130um nozzle.
Software	BD FACSDiva and FlowJo
Cell population abundance	At least 10.000 PE+ and 10.000 APC+ hepatocytes sorted from each animal. The validation was performed by mRNA extraction and qPCR for specific cell markers (Glul for APC+ and Cyp2f2 for PE+ cells).
Gating strategy	FSC-A and SSC-A intial gating was used to exclude debris and cell clumps. Positive single cells were selected with FSC-W and FSC-A gating. Dapi negative cells were selected with BV421 and FSC gating and sorted.<br>For the sc-RNAseq PI negative cells were selected with FSC and PE gating.

☒ Tick this box to confirm that a figure exemplifying the gating strategy is provided in the Supplementary Information.
