# Supplementary material for: Spatial and single-cell profiling of the metabolome, transcriptome and epigenome of the aging mouse liver
Source: Nat Aging. 2023 Nov 9;3(11):1430–45. doi: 10.1038/s43587-023-00513-y (PMC10645594; doi:10.1038/s43587-023-00513-y)
Supplement: Supplementary file 3 — Sequencing metrics. [file 43587_2023_513_MOESM3_ESM.pdf]

Data tables single cell datasets:

Spatial sequencing:

Dataset	# of spots under Tissue	Mean Reads per Spot	Median Genes per Spot
Young 1	1.596	226.296	2.929
Young 2	563	363.437	4.208
Old 1	1.488	182.380	3.138
Old 2	1.846	186.812	3.619

scATAC:

Dataset	Estimated number of cells	Median high-quality fragments per cell	Fraction of transposition events in peaks in cells
Tissue young and old combined I	8.286	21,557	64.4%
Tissue young and old combined II	4.767	27,028	63.7%

SMART-seq3:

Nuclei	Age	Cells	Genes
2n	Old	173	1464
2n	Young	103	3447
4n	Old	136	4312
4n	Young	95	3945
8n	Old	19	4060

8n	Young	19	4217
